# Assessing Lifestyle Patterns and Their Influence on Weight Status in Students from a High School in Sibiu, Romania: An Adaptation of ISCOLE Questionnaires and the Child Feeding Questionnaire

**DOI:** 10.3390/nu16101532

**Published:** 2024-05-20

**Authors:** Mihai Octavian Negrea, Gabriel Octavian Negrea, Gabriela Săndulescu, Bogdan Neamtu, Adelaida Solomon, Mirela Livia Popa, Oana Stoia, Carmen Daniela Domnariu, Minodora Teodoru

**Affiliations:** 1 Clinical Medical Department, Faculty of Medicine, “Lucian Blaga” University, 550024 Sibiu, Romania; mihaioctavian.negrea@ulbsibiu.ro (M.O.N.); bogdan.neamtu@ulbsibiu.ro (B.N.); adelaida.nuta@ulbsibiu.ro (A.S.); liviamirela.popa@ulbsibiu.ro (M.L.P.); oana.stoia@ulbsibiu.ro (O.S.); minodora.teodoru@ulbsibiu.ro (M.T.); 2 County Clinical Emergency Hospital of Sibiu, 2–4 Corneliu Coposu Str., 550245 Sibiu, Romania; 3 “Gheorghe Lazăr” National College, 1–3 Gheorghe Lazăr Str., 550165 Sibiu, Romania; gabriela.sandulescu@cnglsibiu.ro; 4 Department of Clinical Research, Pediatric Clinical Hospital Sibiu, 550166 Sibiu, Romania; 5 Department of Dental Medicine and Nursing, Faculty of Medicine, “Lucian Blaga” University, 550024 Sibiu, Romania; carmen.domnariu@ulbsibiu.ro

**Keywords:** adolescent obesity, family and school environment, questionnaire validation

## Abstract

The escalation of global obesity is driving research to understand environmental influences on this process, particularly during vulnerable developmental stages such as childhood and adolescence. Efforts include the development of various structured data collection tools. We aimed to adapt a series of previously validated questionnaires from the International Study of Childhood Obesity, Lifestyle and the Environment (ISCOLE), the Child Feeding Questionnaire, and elements from the World Health Organization Childhood Obesity Surveillance Initiative (COSI) in order to assess local lifestyle patterns among Romanian high school students and their families that may predispose them to obesity. To this goal, an expert committee was formed as part of a research partnership to oversee the questionnaire’s translation and adaptation. It consisted of education and school management specialists, clinical research professionals, language experts, and public health experts. The adapted questionnaires were then applied to 114 students enrolled in the 9th and 10th grades attending a high school situated in Sibiu, and their parents. The variables measured were investigated for correlations with overweight and obesity and, as a secondary objective, academic performance. The study revealed several critical findings, including suboptimal sleep durations and physical activity levels among students, a significant amount of screen time, and correlations between weight status and physical activity, sedentary time, and maternal weight status and education levels. The adapted questionnaires proved to be effective tools in capturing the multifaceted factors implicated in adolescent obesity, providing a foundation for targeted interventions and broader public health strategies to address this issue.

## 1. Introduction

The global obesity pandemic is continuously expanding [1,2,3]. Constant efforts are being made to decipher the reasons behind this increase in order to devise effective countermeasures. In 1994, Dietz et al. postulated a theory according to which the driving factor leading to the development of obesity is the influence of the environment upon a genetically predetermined background, particularly during certain periods of extreme vulnerability to these factors [4]. Consequently, these particular conditions would be crucial toward the phenotypical expression of weight excess. 

Adolescence stands out as a key developmental phase in this regard [5,6]. In particular, the onset of puberty entails a series of functional and physical changes that have a significant impact on general and psychological well-being, including complex processes concerning sexual maturation. These changes give rise to substantial differences between genders regarding adipose tissue content and distribution [7], physical activity patterns [8], and more intricate concepts such as gender identity, all of which are molded by specific cultural, psychological, and social influences. Exposure to detrimental behaviors concerning dietary intake and physical activity become more potent during this transition period, defined by a volatile state bordering behavioral immaturity and the aspiration of undertaking more personal responsibility. The desire for acceptance and integration into one’s social and cultural environment confers a particular vulnerability for teenagers towards unhealthy habits as promoted by their peers and the surrounding environment. 

Adolescent obesity is of particular interest due to an increased chance of its persistence into adulthood [9]. In consequence, isolating the environmental factors that either initiate, aggravate, or lead to the persistence of weight excess during adolescence is crucial in the attempt to control the global rise in obesity.

One approach in this regard aims to define dietary and physical activity patterns along with various other exposures related to weight increase by using specific questionnaires applied to target populations. An example is the child feeding questionnaire (CFQ), developed in the early 2000s by Birch et al. [10], which aimed to identify familial and parental influences on children’s weight status, encompassing their perceptions, concerns, and practices regarding their child’s nutrition. Though initially intended for prepubescent children, the CFQ’s applicability has also been validated in adolescents [11], offering insights into parental influence during a transition toward greater autonomy.

Another example is the International Study of Childhood Obesity, Lifestyle and the Environment (ISCOLE) [12] which was a massive effort designed to provide a comprehensive overview of environmental factors linked to obesity across 12 different countries with varying income and quality of life profiles from five distinct geographic regions of the world. The study included a series of well-documented and curated questionnaires targeted toward 9–11-year-olds, their parents, and their attending schools’ administration, coupled with several objective measurements such as anthropometry, bioelectrical impedance, and accelerometry. The large collected dataset led to an impressive number of publications shedding light on crucial aspects regarding the influence of lifestyle on childhood obesity [13]. 

Finally, global campaigns such as the Childhood Obesity Surveillance Initiative developed by the World Health Organization, which came as a response to the need for documenting environmental risk factors for obesity in primary school children, are underway as well [14]. COSI was conceptualized as a continuous process from its inception in 2007 and is now undergoing its sixth data collection round during the 2021–2023 school years. It had amassed 45 participant countries by its fifth round, starting from 13 participants during its first [15].

One of the main advantages of these protocols consists of providing a validated method for gathering information based on self-reported data. This allows for result comparison both transversally, across different populations and cultural backgrounds, as well as longitudinally, demonstrating the evolution of lifestyle risk factors in time. Regarding this final aspect, global initiatives such as COSI can significantly increase knowledge by implementing periodic data collection. Although the initial ISCOLE study and the CFQ validation studies were all transversal in nature, they provided a validated framework for questionnaire-based data collection, potentially enhancing the methodological rigor of smaller studies implementing similar data acquisition techniques. 

The present study aimed to provide an overview of local obesogenic lifestyle patterns among high school students using adaptations of previously internationally validated data collection tools. Implementation in a single particular setting has the potential of unveiling strategic interventions tailored precisely for the studied population. At the same time, the propagation of the newly validated tools adapted for the Romanian context can provide a basis for transversal and longitudinal comparative analysis at a national level. As a secondary goal, we sought to investigate which lifestyle behaviors might influence school performance.

## 2. Materials and Methods

### 2.1. Study Setting

This study included adolescents enrolled in the 9th and 10th grades attending a high school situated in Sibiu, one of the main cities in the Transylvanian region of Romania. The study was conducted as part of a research partnership between the high school administration and the Research Department of the Pediatric Hospital of Sibiu. It served as a pilot exploratory study for a set of newly developed data collection tools, adapted from validated questionnaires which were designed to assess lifestyle patterns in children. The study protocol was approved by the hospital’s ethics committee. Candidates for the study were given a consent form by their homeroom teachers with instructions on how to proceed if they wanted to participate. The forms contained details regarding the study’s objective, the methodology involved, and information regarding data use and GDPR compliance. They were reviewed by the legal representative of the Pediatric Hospital of Sibiu.

The consent forms were filled out and signed by the participants’ legal guardians if they wanted to enrol in the study. 

Data were collected using two questionnaires, one to be completed by each student included in the study and one by a student’s legal guardian. The surveys contained questions translated and adapted from the childhood feeding questionnaire, the ISCOLE Demographic and Family Health Questionnaire, the ISCOLE Diet and Lifestyle Questionnaire, part of the ISCOLE Neighborhood & Home Environment Questionnaire [12], the Child Feeding Questionnaire developed by Birch et al. [10] and the WHO European Childhood Obesity Surveillance Initiative (COSI) [14]. After signing the consent forms, the legal guardians were invited to complete the questionnaires addressed to them in printed form. The students involved in the study completed their respective questionnaires using Google Forms, an online tool used for questionnaire-based data collection. Data were collected between the 22nd and 28th of May 2023.

### 2.2. Adapting the Questionnaire

The process of translating and adapting the questionnaire was designed to preserve the validity of the original tools. As in our previous work [16], we followed established protocols for culturally adapting recognized healthcare questionnaires [17,18]. The subsequent steps detail this process.

#### 2.2.1. Assembling the Expert Committee 

To ensure the questionnaires were appropriately adapted, a collaborative endeavor was initiated employing a research partnership between the Pediatric Clinical Hospital of Sibiu’s Research Department and the “Gheorghe Lazăr” National College of Sibiu. This partnership was further expanded to include contributions from faculty members of the “Lucian Blaga” University of Sibiu’s Faculty of Medicine, incorporating expertise from a public health specialist and clinicians affiliated with the County Clinical Emergency Hospital of Sibiu. Moreover, the project engaged a certified local translation agency and an independent Romanian native proficient in English, who possesses a Ph.D. in English and serves as an assistant professor at Shaw University in North Carolina.

#### 2.2.2. Adaptation and Translation

The questionnaire’s adaptation was executed to enhance its relevance and applicability. This task, along with the initial forward translation, were undertaken by the first author, M.O.N. (Ph.D. in medicine and cardiology resident), together with G.O.N., the principal, and G.S., the assistant principal, of the “Gheorghe Lazăr” National College of Sibiu. The initial translation was subsequently refined for clarity and practicality with the assistance of B.N,. from the Department of Clinical Research at the Pediatric Clinical Hospital of Sibiu, and C.D.D., a university professor of public health at the “Lucian Blaga” University. To ensure the translation’s accuracy, a backward translation was performed by two certified translators from a local agency, who were not briefed on the original questionnaires or the study’s aims. Both backward translations are included in the Supplementary Materials (Scan S1: Backward translation 1 and Scan S2: Backward translation 2). A comprehensive review of the initial questionnaire, alongside its forward and backward translations, was conducted by an external expert: a native Romanian and an assistant professor of English at Shaw University, North Carolina, holding a Ph.D. in English, to confirm the fidelity of the translations to each other and the original content, making adjustments where necessary.

The final questions for children and their parents, as well as the corresponding items adapted from the ISCOLE, COSI, and CFQ questionnaires, are included in the Supplementary Materials, Table S1, with changelogs of the modifications made to each questionnaire included in the Supplementary Materials Table S2. The data collected were structured as presented in the following sections, with supporting information from the literature concerning data management methodology.

### 2.3. Data Collection and Structure

The following data were collected either from school records or from the questionnaires implemented.

#### 2.3.1. Child Demographics

For record keeping, we collected the names of the participants enrolled. The use of personal data was thoroughly documented in the consent forms signed by the participants’ legal guardians, wherein none of the personal information gathered by the research team would be made public in any way. Birth dates, gender, ethnicity, and grades of the participants were collected from school records. Age in months was subsequently computed according to the date on which student BMI was calculated. In addition, the country of birth was documented in the questionnaires provided to the legal guardians. The child’s living environment was documented based on the answers in the same questionnaires and also from school records to provide data on the reproducibility of this variable in a manner similar to the one implemented by Castle et al. [19] and then gauge its influence on adolescent obesity [20]. 

#### 2.3.2. Anthropometric Data

Students were asked by their teachers to report their weight in kilograms and height in centimeters. The Body Mass Index (BMI) was calculated using the formula BMI = BW/H^2 [2]. Afterward, BMI Z-scores were determined, employing the 2007 WHO growth references designed for children aged 5 to 19 years [21]. To compute the Z-scores, the WHO AnthroPlus v1.0.4 software was utilized [22], a tool validated in similar previous approaches [23]. Weight category was defined based on WHO criteria, which consider children between 5 and 19 years of age with a measured BMI surpassing the WHO Growth Reference median BMI-for-age by one standard deviation as overweight and those exceeding two standard deviations as obese [3]. Self-reported BMI has been shown to provide acceptable weight category estimation in adolescents and offers an inexpensive and time-efficient method for measuring this parameter [24].

#### 2.3.3. Family Demographics, Health, and History

Data regarding family demographics were collected using adaptations from the ISCOLE questionnaires. They included the age of the participant’s parents, their marital status, the existence of siblings with their respective genders and ages, data regarding the cohabitants of the included student, as well as information regarding the socio-economic status of their legal guardians. In particular, the responding legal guardians’ perception of the family income was inquired, as well as the assets, facilities, and amenities available to their family (number of motorized vehicles, number of television sets, electronic devices available in the household, etc.). Furthermore, legal guardians were queried regarding the highest level of completed education for the biological parents of the participants. Family members who had achieved an educational level equivalent to above International Standard Classification of Education (ISCED) level 4 (University education/Master’s/Doctorate/Postdoctoral studies) were classified as having higher education. Conversely, those who indicated any other category (No completed schooling/Primary education (grades 1–4)/Lower secondary education (grades 5–8)/Upper secondary education/Vocational school/Post-secondary non-tertiary education) were categorized as not possessing higher education. This methodological approach aligns closely with that described by Muthuri et al. [25]. The amount of time spent working outside the home was also documented for both the biological parents and dichotomized as “full time or more” or “less than full time”.

Aspects related to family health included the age of the participants’ biological parents at the time of the survey. The age of the child’s conception was then calculated to investigate possible correlations with the students’ weight status. The weight and height of the biological parents were also inquired. BMI was subsequently calculated, and weight classifications were determined using the established BMI thresholds provided by the Centers for Disease Control and Prevention [26]. Accordingly, individuals with a BMI between 18.5 and 24.99 kg/m^2^ were classified as having normal weight, while those with a BMI of 25 to 29.99 kg/m^2^ were classified as overweight. A BMI of 30 kg/kg/m^2^ or higher was the criterion used to define obesity. The weight status of siblings was also estimated based on the ages and anthropometric data provided in the questionnaires. Appropriate intervals were used for weight category cut-offs corresponding to the age of the siblings, whereby adults were categorized based on the CDC cut-offs mentioned above, and children were categorized using the same principles by which the participants were, by implementing the WHO AnthroPlus v1.0.4 software for Z score approximation [22]. 

Furthermore, the survey included questions regarding the presence of arterial hypertension, diabetes, myocardial infarction or stroke history, peripheral arterial disease, dyslipidemia, or heart failure among the participants’ biological parents as adaptations of similar queries within the COSI.

Inquiries were also made regarding the presence of gestational diabetes during the pregnancy of the participating students. Self-reported gestational diabetes has been previously used and validated to explore its effects on weight status evolution with increasing age [27]. We used a similar approach and phrased the question regarding this aspect by translating the corresponding inquiry from the ISCOLE Demographic and Family Health Questionnaire.

The child’s health history included parameters regarding delivery term, birth weight, and feeding practices during early childhood (i.e., breastfeeding and formula-feeding and their duration). Recalled birth weight has shown acceptable reliability when reported by parent or guardian respondents of adolescents [28]. This parameter was categorized similarly to Qiao et al. [29] using 3500 g and 4000 g as category cut-offs. Birth length was omitted due to this parameter’s known low recall accuracy [30].

As a measure of general well-being, questions dedicated to health-associated quality of life were also used, as in the original ISCOLE questionnaire, where they were based on the Kidscreen-10 index [12,31]. Consequently, a quality-of-life score was computed within our study, similar to the Kidscreen-10 score. The score was calculated based on the instructions provided by the developers of this index [32]. Namely, individual item scores were added up for each question after appropriate coding of answers with values from 1 to 5. The final scores, therefore, could range between 10 and 50.

#### 2.3.4. Physical Activity, Sedentary Time, and Sleep Patterns 

The implemented questionnaires collected data regarding daily physical activity, sedentary time, and sleep duration. Daily screen time was calculated using a method previously validated by Katzmarzyk et al. [33], assigning a numerical value to questionnaire responses as follows: 0 for not watching television or using the computer, 0.5 h for less than 1 h of screen time, and direct quantification for response options of 1, 2, 3, or 4 h. For responses indicating 5 or more hours of screen time, a value of 5 h was assigned. To ascertain the mean daily number of hours spent in front of screens, television and computer time were aggregated, and mean times were calculated using the formula described in the same study mentioned previously as the weighted average of daily hours spent in front of screens on weekdays and weekends: 2/7 * weekend daily average + 5/7 * weekday daily average. The results were compared across participant weight category groups. Average daily outdoor time was computed in a similar manner by using the intervals provided in the questionnaires regarding outdoor time before and after school during weekdays and outdoor time during weekends. In addition, collected data included the number of days in which the respondents participated in physical education classes and the number of days in which they performed a minimum of 60 min of moderate-to-vigorous physical activity (MVPA), defined in the questionnaire as “activities that make your heart beat faster and make you breathe faster than usual”. These questions were adapted from the ISCOLE Diet and Lifestyle questionnaire, and a part of them were, in turn, obtained by the original authors involved in ISCOLE from the Youth Risk Behavior Surveillance System [34].

Active transport to and from school were also documented in the questionnaires, and the data were organized in a similar fashion to Gropp et al. [35], wherein three categories of active transportation were defined. The first category contained students who did not regularly use active transportation (i.e., traveled by personal motorized vehicle or public transportation), the second entailed students who regularly used active transportation by walking or using various self-propelled vehicles (bicycle, scooter, rollerblades, skateboard) and lived close to school (under 5 min by bicycle or other self-propelled vehicle or under 15 min by walking), and the third category was defined by regular use of active transportation used for greater lengths of time than those specified in the second category.

Further aspects concerning media exposure and physical activity were collected by documenting access to certain electronics predisposing towards sedentary time on the one hand and facilities for physical activity available both at home and in the proximity of the enrolled students’ domicile on the other, as well as the degree of parental implication in the students’ physical activities. In addition, several questions documented particular inclinations towards certain activities such as team sports, martial arts, or artistic activities. Psychological attitudes towards physical activity were measured in a similar fashion to previously described methods, by computing scores for intrinsic motivation [36] and self-efficacy [37] related to physical activity. The scores were calculated as sums of the values provided by Likert scales (from 1 to 5) implemented as response options for the questions designed to measure these constructs. This methodology has been described in detail and successfully implemented in past research [38]. For computing the intrinsic motivation score, questions regarding external pressure or lack of motivation (21 and 23 of the original ISCOLE Diet and Lifestyle questionnaire) were reverse-coded.

Sleep duration was computed as the interval between bedtime and wake-up time, as declared in the questionnaires. The methodology implemented aligned with protocols previously described which have shown accurate recall estimates of sleep times from adolescents [39]. Students were then categorized according to whether or not they achieved the minimum recommendation provided by the CDC for adolescents of an 8 h nocturnal sleep interval [40]. School days and weekends were taken separately into account in this respect.

#### 2.3.5. Dietary Patterns and Attitudes

Dietary patterns were recorded using a 23-item food frequency questionnaire (FFQ), adapted from the ISCOLE Diet and Lifestyle Questionnaire, which in turn used a similar tool adapted from the Health Behaviour in the School-Aged Children study [41]. This questionnaire allowed participants to select between categories ranging from “never” to “more than once a day” regarding the weekly consumption of the 23 available food items. In a similar approach to Mikkilä et al. [42], available options were converted into weekly portions. In their original work, “never” was converted to 0, “less than once a week” converted into 0.5, “once a week” into 1, “on 2–4 days a week” to 3, “on 5–6 days a week” to 5.5, “every day” to 7, while “more than once a day” was converted into 10 weekly portions. We used the same conversion strategy. However, in the final version of our adapted questionnaire we removed the option “Never” due to perceived overlap with the option “less than once a week” upon translation and converted the latter option to 0. Principal Component Analysis (PCA) was performed to identify dietary patterns. This approach has been previously implemented on datasets obtained from the FFQ part of the ISCOLE questionnaires [42]. PCA analyses showed consistent results in revealing two dietary patterns—one associated with healthy eating and the other with unhealthy eating. Consequently, we ran PCA in our study with a prespecification of a fixed number of two components in the analysis. The initial phase involved ascertaining the item loadings, followed by the application of a Varimax rotation with Kaiser normalization to enhance the item loadings on the identified components, thus aiding in the elucidation of the data structure. A cut-off of 0.3 was selected for item loading in identifying variables integrated into one of the two patterns, whereby loading on one pattern with a value of above 0.3 and on the other pattern with a value below 0.3 attributed the variable as belonging to the first pattern.

Students were also inquired with regards to food consumption in front of screens. As the answers had the same structure as in the study conducted by Mikkilä et al. [42], they were converted into weekly portions using a similar approach. Other information regarding feeding practices included patterns related to breakfast consumption and meals eaten away from home, as well as data concerning dietary behavior in relation to emotional status. This final aspect was measured using the response to 7 items within the survey, which measured emotional eating. They were graded from 1 to 3, corresponding to options “never or almost never”, “sometimes”, and “usually or always”. An emotion-induced eating score was calculated by summing up the scores of each of the individual 7 answers, as described in previous literature [43].

In addition, several questions sought to collect data regarding the availability of food types in the household, as noted by the participants’ legal guardians, as well as the proximity of sources for food procurement and associated food-shopping habits.

To further document parental attitudes towards child feeding practices, the questions within the Child Feeding Questionnaire were also included in the surveys completed by the legal guardians. Data processing in this regard was implemented in a similar fashion to Birch et al. [10] and Kaur et al. [11], whereby we performed confirmatory factor analysis for the initial seven-factor model proposed with subsequent alterations to improve model fit to parents of adolescents as respondents.

#### 2.3.6. School Performance

General school performance was documented by recording the students’ general averages of the marks obtained in the disciplines studied across the school year once it was completed. In Romanian high schools, a 10-point scale is used for grading each subject, and the average at the end of the school year is calculated as a mean of the grades obtained. We then evaluated correlations between averages obtained by students at the end of 9th and 10th grades with various parameters measured in the questionnaire. 

### 2.4. Preliminary Testing

A preliminary pilot sample was analyzed to detect potential issues regarding the clarity of the questions and identify and rectify ambiguities, complex wording, or confusing questions that could potentially lead to inconsistent responses. Twelve participants and their parents were selected for this endeavor. Participants were chosen outside the school to avoid influencing the target population. In addition, the responders in the pilot sample fell into a broader age interval (8 to 17) to ensure the clarity of our surveys across multiple comprehension levels. The sample size for the preliminary survey was determined based on authoritative sources within the field [44,45]. Reflecting on the insights of Sheatsley P., whose seminal contributions to questionnaire design are well regarded, identifying primary issues and shortcomings in a trial questionnaire generally requires between 12 and 25 participants [46]. After repeated consultations with members of the pilot sample, which were conducted to resolve any potential sources of misinterpretation in the surveys, the final versions of the questionnaires were approved by the expert committee and sent for backward translation. 

### 2.5. Inclusion/Exclusion Criteria

To implement the questionnaires adapted in the process outlined above, a wider pilot exploratory study was conducted. The study included students enrolled in the 9th and 10th grades attending the “Gheorghe Lazăr” National College of Sibiu. Only students who had provided a signed consent form from their legal guardians were allowed to participate in the study. The study did not include adopted children, firstly due to the well-known intricacies of weight status balance in this population with regard to the interplay between genetic background and environmental exposure in the context of the foster family [47], and secondly due to the expected small count of such participants which would have not been suitable for a sub-analysis. Children whose legal guardians signed the consent form for participation but did not complete the survey were excluded from the study. Participants could, however, opt out of answering certain questions if they wished to do so. Notwithstanding, certain datapoints were considered crucial for obtaining an adequate analysis of the risk factors associated with adolescent obesity and were considered mandatory in order to allow for integrated data interpretation. Namely, child anthropometric data, sleep-related data, parental education levels, and weight status were selected as key components, and participants with missing data in any of these categories were excluded. The aforementioned variables were selected based on previously described results which highlighted the importance of these parameters in childhood and adolescent weight balance [48,49]. With respect to other variables known to significantly influence childhood and adolescent weight, such as family income, birth weight, breastfeeding, and sibling weight status, pairwise deletion was implemented as a compromise for data retention, and subgroup analyses were performed. 

### 2.6. Statistical Analysis

Categorical data were represented through the utilization of frequency counts and proportion metrics. Metrics such as mean values, standard deviations, and ranges—including minimum, maximum, and interquartile values—along with 95% confidence intervals for mean estimates characterized continuous variables. The distribution normality of continuous data was evaluated via the Kolmogorov–Smirnov and Shapiro–Wilk tests, as well as the evaluation of skewness and kurtosis and visual inspection of box plots. For group comparisons, categorical variables were analyzed using Chi-square or Fisher’s exact tests as appropriate, whereas continuous variables adhering to a normal distribution were assessed using independent sample t-tests to discern mean differences between two groups. In cases where continuous variables deviated from normal distribution, the Mann-Whitney U test was utilized. For comparisons involving three or more groups, normally distributed data were analyzed with one-way ANOVA, while the Kruskal–Wallis test was applied to data with non-normal distributions. In situations involving paired samples with non-normally distributed data, the Wilcoxon signed-rank test was administered. All statistical tests were conducted with a significance level set at α = 0.05. Software used for data analysis included Microsoft Excel v 2402, SPSS v.21, and R v 4.3.3.

### 2.7. Questionnaire Validation

An investigation into the questionnaire’s construct validity and internal consistency was conducted on items that utilized uniform non-binary response scales, where the responses could be affected by the participants’ subjective views. The measurement of internal consistency was undertaken through the calculation of Cronbach’s alpha. The interpretation of this parameter was conducted in accordance with previous guidelines in this respect [50] and values above 0.7 were considered acceptable.

With regard to fit statistics when performing confirmatory factor analysis, the metrics computed were chi-square statistics (χ^2^/df and statistical significance), comparative fit index (CFI), Tucker–Lewis index (TLI), Root Mean Square Error of Approximation (RMSEA) and Standardized Root Mean Square Residual (SRMR). Cut-offs for these variables indicating acceptable model adjustment to the data were derived from authoritative literature in this regard [51,52]. Namely, a value below 2 for χ^2^/df was considered indicative of an acceptable fit regardless of a statistically significant chi-square statistic, and values of 0.9 or above were deemed satisfactory for CFI and TLI. Values below 0.08 for RMSEA and SRMR were also interpreted as representing an adequate fit to the model. These benchmarks have been successfully utilized in previous endeavors of translating and adapting questionnaires aimed at children with a similar demographic to our study [53].

**Figure 1:**
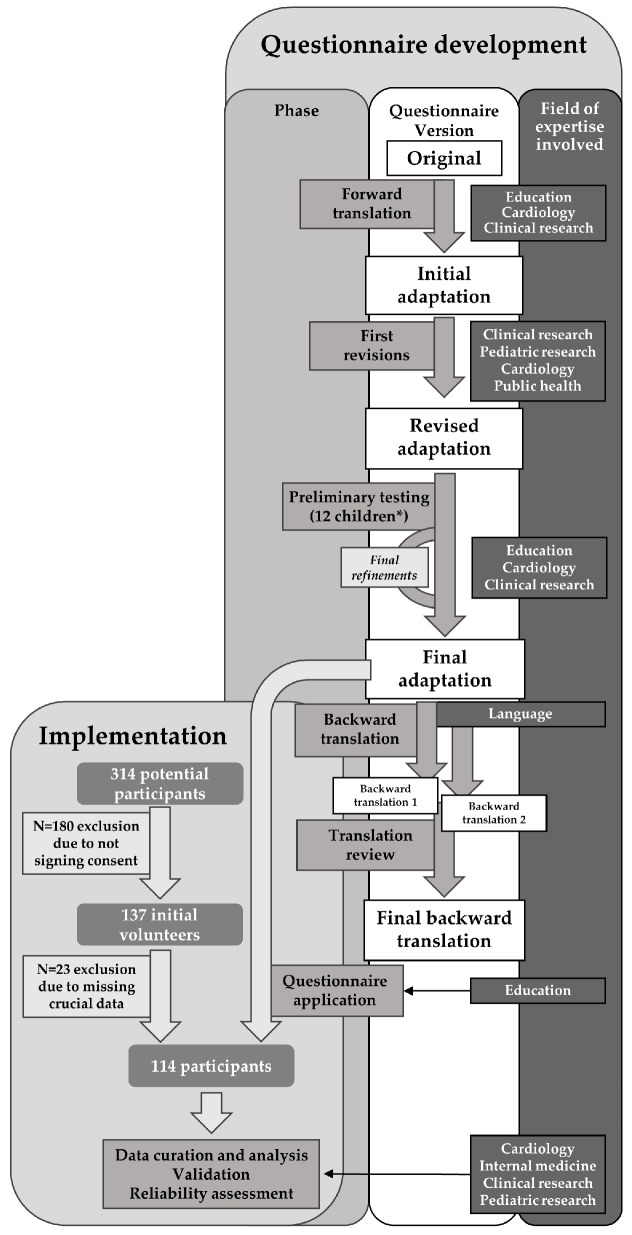
Questionnaire development and implementation workflow and field of expertise of contributors involved in each phase. Authors are represented by use of their initials, experts mentioned in the acknowledgement section are represented by use of their full names. * The 12 children included in preliminary pilot testing, ranging in age from 8 to 17, were not students at the high school where the study was conducted. Data derived from the questionnaires completed by these 12 children and their parents were not included in the study, as this phase served only for enhancing the clarity of the items in the questionnaire.

Figure 1 provides an overview of the methodology and workflow utilized in questionnaire development and implementation, showcasing the experts involved in each phase of the project.

## 3. Results

### 3.1. Demographic Data

Of the total 314 9th- and 10th-grade students enrolled at the “Gheorghe Lazăr” National College, 137 participants (43.63%) provided signed consent forms from their legal guardians. Of these, 23 participants were excluded due to missing data regarding student BMI (n = 4), sleep times (n = 1), parental weight status (n = 13), education levels (n = 4), or not completing the survey for the legal guardian at all (n = 1). 

The final sample included 67 girls (58.77%) and 47 boys (41.23%) with ages between 185 and 211 months (mean ± standard deviation (SD): 197.48 ± 6.63) with no significant differences concerning age across genders. 

### 3.2. Anthropometric Data and Descriptive Statistics

**Figure 2:**
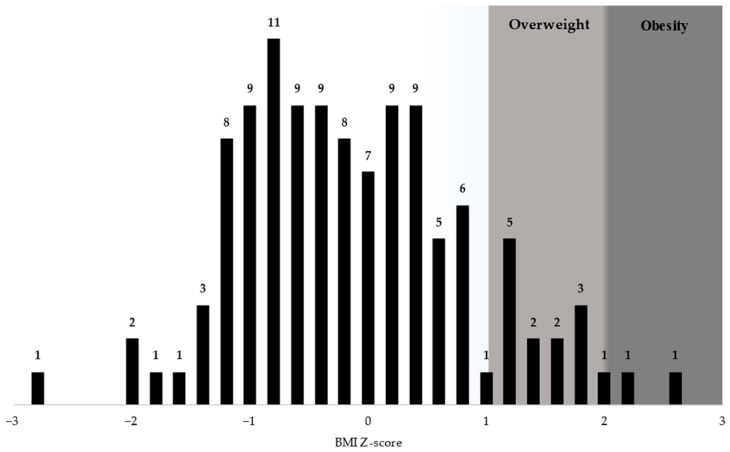
BMI Z-score distribution for students.

BMI Z-scores were normally distributed among the students enrolled, with a mean of −0.24 and a standard deviation (SD) of 0.98. Of the studied sample, 15 children (13.2%) had a BMI Z-score above one. Within this subgroup, 13 children (11.4% of the total 114) were overweight, and 2 students were obese (1.75% of the total). The distribution of BMI Z-scores for the students enrolled as well as weight status classification within the distribution are presented in Figure 2.

Based on self-reported student BMI and parent perception of the child’s weight status at the time of questionnaire completion (as inquired within the CFQ) we could determine the accuracy of this perception. Normal weight students had their category perceived correctly by their parents in 93.94% of cases, while parents of students with weight excess underappreciated their children’s weight status in 61.54% of cases for overweight students and 50% of cases for obese students. A graphical representation of these results is presented in the Supplementary Materials Figure S1.

**Figure 3:**
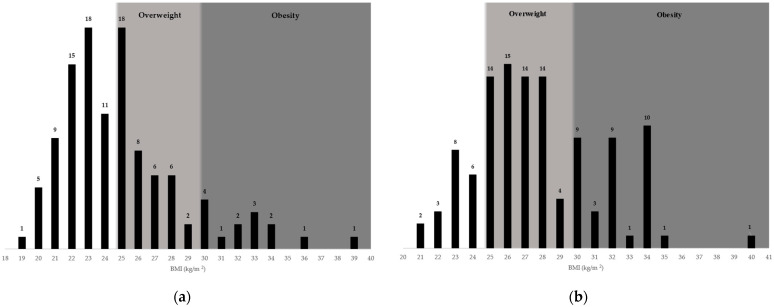
BMI distribution of (**a**) mothers and (**b**) fathers of students enrolled.

Among the students’ parents, overweight was present in 24.6% of the respondents’ mothers and 49.1% of the fathers, while obesity was present in 9.6% of the mothers and 21.9% of the fathers. The differences observed were statistically significant. The distribution of BMI for the parents of the students enrolled, as well as weight status classification within the distribution are presented in Figure 3.

In our sample of 114 participants, 103 (90.4%) of the questionnaires completed by the legal guardians were filled in by the students’ mothers, while 9 (8.8%) were completed by fathers and 1 (0.9%) by a student’s stepfather.

**Figure 4:**
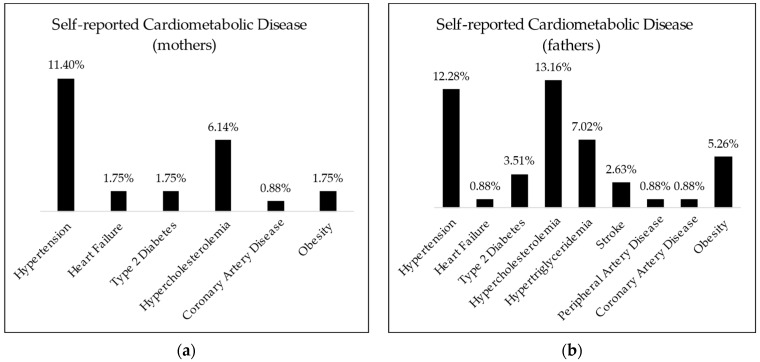
Declared cardiometabolic diseases of (**a**) mothers and (**b**) fathers of students enrolled.

Based on the answers obtained from the CFQ regarding parents’ self-perception of weight, 91.55% of normal weight mothers correctly perceived their weight category, with the remainder overestimating it as being overweight. Conversely, 34.78% of overweight mothers and 77.77% of obese mothers underestimated their weight category. A more detailed representation of these aspects is illustrated in the Supplementary Materials, Figure S2. Only two of the included mothers declared having obesity as a disease, even though their calculated BMI categorized them as overweight. Other metrics of reported family cardiometabolic disease are presented in Figure 4.

**Figure 5:**
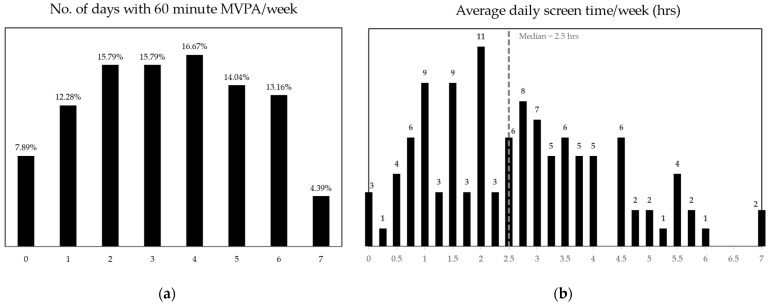
Distribution of weekly MVPA (**a**) and average daily screen time (**b**).

Data concerning the allocation of time across sleep, screen time and physical activity patterns indicated that only 28.1% of responding students achieved a minimum of 8 h of sleep during school days. The distribution of self-reported 60 min moderate-to-vigorous daily physical activity and that of average time spent in front of screens during an entire week are presented in Figure 5.

With regard to screen access, 100% of the students included had access to mobile phones, and 93.9% of students had access to a computer or television in their bedroom.

### 3.3. Factors Associated with Adolescent Overweight and Obesity

Variables derived from the questionnaire were extensively studied to highlight their relationship with student weight status. A selection thereof, including the variables showing significant correlations are presented in the following sections.

#### 3.3.1. Demographic and Socio-Economic Factors

Table 1 lists a series of categorical demographic and socio-economic factors and their relationship with body weight category within our study group.

There were no statistically significant differences regarding age across weight categories. Marital status, cohabitation with particular family members (biological father, grandparents, etc.), or time spent working outside the home by parents were not significantly correlated with respondent weight status. Neither were resources such as available electronic devices (phone, television, tablet, etc.). Further variables related to socio-economic status, including the number of vehicles available to the family, and the number of televisions in the household also showed no significant correlations with weight category.

The declared living environment also did not show any correlation with weight status. The overall accuracy of this response when compared to data extracted from school records was 88.5%, with urban dwellers significantly more likely to misclassify their living environment compared to rural dwellers (12.5% vs. 5.88%, *p* < 0.01).

#### 3.3.2. Child and Family Health History

With regards to data concerning child and family health history, several categorical variables and their relationship to body weight of the students enrolled are presented in Table 2.

Furthermore, sibling weight status was not associated with the weight category of enrolled students. 

Table 3 showcases a selection of the investigated numerical variables quantifying aspects related to child and family health history and their distribution across student weight categories.

In addition to these results, general health perception did not correlate with body weight category.

#### 3.3.3. Physical Activity, Sedentary Time, and Sleep Patterns

Data regarding the distribution of categorical variables related to physical activity, sedentary time, and sleep patterns across student weight categories are presented in Table 4.

Data regarding the distribution of numerical variables related to physical activity and sedentary time across student weight categories are presented in Table 5.

Propensity for specific forms of physical engagement, such as team sports, martial arts, and dance, alongside the availability of electronic devices that may contribute to sedentary behavior or the presence of exercise amenities within the domicile, exhibited no substantial correlation with weight status as measured by BMI Z-score. Neither did the degree of parental implication in the students’ physical activities or the exact context or location in which physical activity was engaged by the students (at home, in parks, etc.).

With regard to the amenities present in the vicinity of the participants’ homes, a single item showed a significant correlation to student weight status, namely access to public spaces with sports equipment. The results are shown in Table 4.

In addition, the perceived adequacy of sleep, both in terms of quality and quantity, as reported by the participants, manifested no notable correlation with weight category.

#### 3.3.4. Dietary Patterns and Attitudes

**Figure 6:**
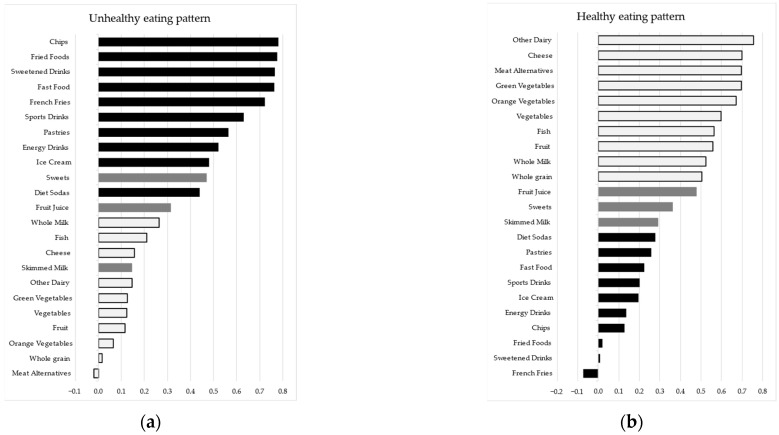
Factor loadings for weekly portions of 23 food types on components obtained from PCA: (**a**) Component 1—Unhealthy eating pattern, (**b**) Component 2—Healthy eating pattern. Variables with a loading of 0.3 or above on component 1 and below 0.3 on component 2 (i.e., mainly loading on the unhealthy eating pattern) are marked by solid black fill, while variables with a loading of 0.3 or above on component 2 and below 0.3 on component 1 (i.e., mainly loading on the healthy eating pattern) are marked by light gray fill and black outline. Items left with dark gray fill either loaded significantly (≥0.3) or insufficiently (<0.3) on both components.

To assess dietary patterns in our study group, we performed PCA analysis with varimax rotation, with the pre-specification of a fixed number of two principal components. The individual factor loadings of each variable included in the FFQ after Varimax rotation with Kaiser Normalization are represented in Figure 6.

Component 1 (unhealthy eating pattern) explained 21.15% of the model variance, while component 2 explained 20.7% of the model variance.

We computed total weekly portions of healthy and unhealthy foods as identified by PCA and compared these totals across weight categories. We performed similar comparisons for each food group in the FFQ as well as concerning the number of weekly portions of food consumed in front of screens. The comparisons regarding total weekly healthy and unhealthy foods, the food groups within the FFQ with significant differences regarding the number of weekly portion consumption (from the FFQ and in front of screens) across weight categories, and the Emotion-induced eating score are all presented in Table 6. In addition, Breakfast consumption and lunches eaten at school or weekly meals eaten outside the home did not significantly correlate with weight status as measured by BMI Z-score in our sample.

Within our study sample, 109 legal guardians provided complete responses for the Child Feeding Questionnaire. We conducted a confirmatory factor analysis to test the fit of the initially proposed seven-factor model to our data (with the error covariances suggested in the initial study). In this initial analysis, we did not include the question we added to the questionnaire (after question 28—see Supplementary Materials, Tables S1 and S2). The analysis yielded a significant Chi-square test statistic, indicating a significant difference between the observed and predicted covariance matrices (χ^2^/df = 3.8; *p* < 0.01). The Comparative Fit Index (CFI) was 0.822 and the Tucker–Lewis Index (TLI) was 0.788. The Root Mean Square Error of Approximation (RMSEA) was 0.074, with a 90% confidence interval ranging from 0.060 to 0.087. Furthermore, the Standardized Root Mean Square Residual (SRMR) was 0.084.

This initial fit was acceptable from the viewpoint of RMSEA but unsatisfactory regarding the other fit parameters. Previous work suggested the possible existence of error covariance between variables concerning perceived childhood weight in the past as well as between variables concerning perceived adulthood weight in the past. Consequently, we adapted the current model to reflect this by specifying error covariances between all the variables concerning perceived child weight pertaining to the past. The initial study essentially used the same approach; however, their target group only included younger children and, therefore, used fewer categories concerning perceived child weight in the past. Our implementation adapted this approach to using five categories related to perceived child weight in the past.

Furthermore, the combined item concerning food rewards (RST3) showed low loadings on all factors in the initial seven-factor model. Therefore, it was removed from our current model.

The modifications mentioned led to a model with improved fit indices, where the CFI increased to 0.921 and the TLI improved to 0.900. The RMSEA further decreased to a level of 0.052, with a 90% confidence interval from 0.033 to 0.069. Additionally, SRMR decreased to 0.076, further confirming the model’s adequacy. The Chi-square statistic remained statistically significant (*p* < 0.01), however, the χ^2^/df decreased to 1.3.

Adding the newly inserted question either to the “concern” factor or the “restriction” factor marginally reduced fit indices (CFI = 0.892, TLI = 0.867, RMSEA = 0.06 CI 90%—0.043–0.074; SRMR = 0.079 for adding to “concern”, CFI = 0.917, TLI = 0.897, RMSEA = 0.052 CI 90%—0.034–0.068; SRMR = 0.077 for adding to “restriction”). The results indicated, however, that this item was better suited to be regarded as a restriction factor.

After obtaining a model with a satisfactory fit as outlined above, we proceeded to evaluate the correlation between the metrics measured by the adapted CFQ, both individually and in combinations aligning to the measured constructs of each parameter as sums. Significant correlations with student weight status are presented in Table 7.

Food types available in the household did not significantly correlate with weight category. Mean BMI Z-score was, however, lower in students whose parents declared the availability of “other sweets” more than “sometimes” in their household (mean ± SD: −0.47 ± 0.97 vs. 0.07 ± 0.92; *p* < 0.01). 

Students’ weight status did not significantly correlate with their legal guardians’ declared proximity to the home of sources for food procurement. Parents’ food-shopping habits and perceptions also did not significantly correlate with student weight status.

### 3.4. Factors Associated with School Performance

We investigated correlations between the average marks of the students in 9th and 10th grade and various parameters measured in the questionnaire. The main results are presented in the Supplementary Materials, Table S3. Values were normally distributed across all categories investigated, except for 10th grade students with high estimated family income.

Students in the 9th grade who slept at least 8 h every night during school days had significantly higher grade averages compared to their colleagues which did not attain this benchmark (mean ± SD: 9.56 ± 0.3 vs. 9.37 ± 0.35; *p* = 0.038). 

### 3.5. Questionnaire Internal Consistency

We evaluated the questionnaire’s construct validity and internal consistency for items featuring similar, non-dichotomous response scales, where the responses could be affected by the participants’ subjective views. The measurement of internal consistency was undertaken through the calculation of Cronbach’s alpha. The results are presented in Table 8.

## 4. Discussion

Our adaptation of the ISCOLE and CFQ questionnaires with elements from the COSI questionnaire provides a valuable tool in standardized data collection within the Romanian context, offering insights into their obesogenic lifestyle practices and attitudes. The internal consistency of the adapted questionnaires demonstrated satisfactory results, highlighting the reliability of the data collected and its subsequent analysis. Particularly, the outcomes revealed concerning lifestyle patterns among the children studied, including excessive screen time, inadequate physical activity, and insufficient sleep when compared to the current CDC guidelines.

In addition, several correlations between student weight status and the variables measured by the newly developed tools were observed. A summary of these findings is illustrated in Table 9.

### 4.1. General Findings

From the data collected, several key aspects can be deduced regarding the lifestyle of adolescents enrolled in the “Gheorghe Lazăr” National College.

Particularly, less than a third of the students enrolled in our study achieved the nightly sleep recommendations of the CDC of at least 8 h during school days. Furthermore, less than 5% of the students reported achieving the CDC-recommended daily 60 min of moderate-to-vigorous physical activity [57] during a week, and more than half of them spent a daily average of at least two and a half hours in front of television or computer screens across an entire week.

Regarding weight status, although the BMI Z-score had a normal distribution in students, with the proportions of overweight and obesity not exceeding that of an expected Gaussian curve within this age interval, weight excess showed significantly higher prevalence in parents. This is particularly concerning within the context of the low awareness of this aspect, as revealed by how mothers completing the questionnaire perceived their own weight. The same low awareness is what might be incriminated for the low prevalence of self-declared cardiometabolic diseases. This aspect has been previously thoroughly documented within young Romanian adults [58].

In consequence, while the prevalence of self-reported overweight and obesity among the students enrolled may not have strayed from normal expectations, our data suggest a much broader risk burden coming from within the family health environment. Further strengthening this aspect is the correlation found between various factors and student weight status, with an evident proclivity towards maternal traits. Namely, maternal obesity was strongly associated with student overweight and obesity. The definitory role of maternal adiposity on child weight balance has been thoroughly documented in previous literature [55]. 

The association found in our study between the absence of maternal higher education and student weight excess further reinforces the maternal role in child development. Previous findings have reported varying relationships in this regard, including the influence of various covariates, including family income and higher-order correlates such as the country’s human development index [13]. Paternal metrics did not, however, correlate with child weight status. Gender disparities were also evident in the distribution of weight excess, wherein males were more likely to be overweight or obese, both regarding students and their parents, aspects which have also been previously described in the literature when considering sex disparities regarding weight excess, particularly in developed countries [54,59]. 

The association found in the current study between elevated birth weight and subsequent adolescent obesity has also been well documented in previous literature [29]. This parameter is essentially a complex indicator, revealing genetic predisposition on the one hand while encapsulating the overarching influence of the familial background on the other [60]. This final aspect includes lifestyle habits and exposures that likely begin to exert their influence in utero and continue to do so by subsequent exposure in the home environment, with some studies suggesting that antenatal dietary intervention may reduce subsequent predisposition toward childhood obesity, without there being a clear consensus in this regard however [61].

In the attempt to investigate which particular traits of this home environment have an obesogenic influence, it becomes apparent that significant transformations have occurred in this area, reflecting the current era’s technological evolution. In our study, all of the students enrolled had personal access to a mobile phone, and more than 90% of them had access to either a computer or a television in their bedroom. This distribution of ubiquitous exposure to screens is not an anomaly but rather the current norm of modern times and stands as a hallmark of the remarkable technological advancements of the past few decades. The presence of screens in adolescents’ daily lives has transitioned from being a casual element to a critical aspect of their environment, necessitating a deliberate approach to managing screen time. Judicious screen exposure in this context has shifted towards being a learned behavior rather than a spontaneous one, with the ultimate goal of balancing the known beneficial and deleterious effects of screen exposure [62]. Within the current study, the association between increased screen exposure (predominantly through television watching) in our sample and increased body weight is a further argument favoring a balanced approach toward the use of electronic devices.

With regard to dietary exposures, our data are in alignment with previous findings of the ISCOLE initiative, wherein through the analysis of the responses provided, two major nutritional tendencies have emerged—one inclined towards healthier foods and one towards unhealthy ones [42]. One particular element that is unaccounted for and has arisen in our study is a declared tendency of overweight students to consume fewer portions of potato chips compared to normal-weight students—both overall during the week as well as in front of screens. This correlation may be due to social desirability bias inherently present in studies of this type. It may denote, however, one key aspect—which is that the students enrolled in our study may possess a nuanced understanding of nutrition, being aware of which food groups are particularly obesogenic and harmful. Despite this knowledge, the persistent consumption of such foods suggests a complex interplay between awareness and behavior. This contrast may highlight potential gaps between nutritional education and its practical implementation in daily dietary habits. It stresses the need for measures that do more than simply inform but also support the application of this understanding to foster better eating practices, especially in settings where unhealthy food options are widespread and readily available.

Regarding parental involvement in adolescents’ dietary habits, our application and modification of the Child Feeding Questionnaire have reinforced previously established theories and enhanced our comprehension of parental dietary impacts on Romanian adolescents. Notably, after several modifications, the final version of the translated questionnaire conformed to the seven-factor model initially proposed by Birch et al. [10]. Particularly, our findings suggest that parental restrictive practices did not accurately represent their intended construct in adolescent populations. This observation is consistent with the research of Kaur et al. [11], where, in their questionnaire adaptation for adolescents, the item RST3 (pertaining to food as a reward) was dropped from the final model. This exclusion may stem from adolescents’ particular resilience to such influences amid growing autonomy. A further aspect that was also documented by Kaur et al. was the persistence of significant chi-square statistics, indicating a notable difference between the observed and predicted covariance matrices despite otherwise satisfactory fit indices. This issue might be linked to the sample size or further necessary refinements and points to the need for additional testing of the CFQ.

Nonetheless, our final model obtained values below 2 for χ^2^/df, which is, as mentioned in the cited literature, representative of an acceptable model fit regardless of the significant chi-square statistic. In essence, our results align with those of Kaur et al. regarding the use of the CFQ in adolescents and provide a strong foundation and a preliminary model for more in-depth future studies, even more so considering the significant associations between parental attitudes and child weight—namely regarding concern, monitoring, and perceived weight, but also with respect to some restrictive tendencies (i.e., regarding sweets), which warrant further investigation.

When expanding beyond the family context, certain findings described in the previous literature were absent in our sample, such as the link between breakfast consumption and body weight category [63]. This discrepancy is most likely attributable to the relatively small sample size of our study. Notwithstanding, some outcomes mirrored those documented in existing research on elementary school students, such as the correlation between regular participation in physical education (PE) classes and weight status [56]. Additionally, our research described other interesting associations, such as the link between lower body weight category and easier access from home to public spaces with sports equipment. This suggests that the availability and characteristics of physical activity opportunities may play an important role in shaping body weight and could inform future public health initiatives and urban planning strategies to enhance physical activity accessibility.

### 4.2. Questionnaire Validation

Finally, concerning the validity of the tools and instruments developed in our research, the internal consistency of the adapted questionnaires, as indicated by Cronbach’s alpha values, suggested generally satisfactory measurements. Several sections demonstrated good internal consistency, such as the self-efficacy for PA section and the monitoring section within the CFQ. There are, however, exceptions to this trend, with certain sections indicating lower reliability. Specifically, the ‘Perceived Parent Weight’ section of the CFQ (Child Feeding Questionnaire) registered a Cronbach’s alpha of 0.438. This may be attributed to the subjective nature of weight perception, which can vary significantly between respondents, leading to inconsistent responses.

Moreover, the ‘Food Shopping’ section showed a particularly low alpha value of 0.275, potentially due to recall bias, as respondents may have struggled to remember and report past shopping behaviors accurately. The same might be true concerning the “Family” section within the ISCOLE-NHEQ, which showed a Cronbach’s alpha of 0.488. Apart from these lower extremes, most of the items exhibited a value of 0.7 or above, showcasing their effectiveness in measuring their designated constructs. In future research, supplementary measures or methodological adjustments may enhance reliability, particularly for constructs that inherently involve subjective assessments or recall-dependent responses.

### 4.3. Overall Achievement of Objectives

Our main goal was to culturally adapt a series of standardized data collection tools for evaluating the lifestyle habits and dietary patterns of Romanian adolescents and their parents.

The primary use of these tools within the current study was to assess these factors in a restricted community. This goal was successfully achieved by collecting data that highlighted alarming trends in these adolescents’ lifestyle choices. Our endeavor was a localized intervention, primarily educational and informational in nature, mainly intended to raise awareness. This evidence-based approach was tailored to present the correlations between lifestyle habits and weight status or academic performance, yielding potentially tangible outcomes of particular interest to the parents of the participating children.

Moreover, the survey instruments were devised with the aim to holistically address health within the family context. In this regard, the low incidence of reported cardiometabolic diseases may be viewed as a decreased awareness of these diseases and may present an opportunity for early intervention and prevention. One example would be the potential dissemination of information regarding the SCORE-2 prediction algorithm as a powerful tool to raise awareness regarding cardiometabolic risk and guide preventive strategies. The European Society of Cardiology currently recommends using this score in apparently healthy people aged between 50 and 69 to estimate cardiovascular disease risk as a screening tool [64]. This fits the demographic of the responding parents who did not declare the presence of cardiovascular diseases, most of whom are either in the targeted age range or on the verge of reaching it.

Furthermore, the developed tools were crafted with the intention to extend their influence beyond the individual and familial structures as well. This objective encompasses both the immediate educational setting and extends to a broader spectrum, including public health interventions. For example, the data suggest the need to improve access to public sports equipment, indicating a potential for municipal or school-level initiatives. These could entail weekend activities designed to engage overweight children, borrowing from successful strategies highlighted in other studies, which we have previously documented in our work [16]. In particular, data regarding student involvement in physical education classes may suggest the potential for initiatives aimed at increasing inclusion and participation. Our data indicated that students who did not participate in PE were significantly more likely to be overweight. This raises the possibility that non-participation in PE may be due to social stigma or a discrepancy between the physical aptitudes required by the existing curriculum and those of the students [65]. Addressing these barriers by adapting PE programs to accommodate diverse body types and abilities, and by fostering an inclusive and supportive environment may have the potential of improving PE participation [66].

Ultimately, the scope of our project extends beyond local application. By making the adapted questionnaires available for broader use, we aim to enable their application on a national level. This would allow for cross-sectional analysis across various settings, as well as longitudinal studies to observe trends over time. The intent is to establish a foundation that can be refined into a practical instrument, assisting policymakers and educators in developing targeted interventions. Such a tool would be adaptable to the unique requirements of diverse groups, potentially improving the impact of health promotion efforts across the country. Data derived from these tools may guide the local employment of strategies available from a vast array of available interventions [67].

### 4.4. Strengths and Limitations

The current study offers several noteworthy strengths in the context of health education and research. Firstly, our approach is designed toward the potential development of tailored health education strategies to address specific community needs based on the pragmatic implementation of knowledge collected in a standardized manner. An additional strength lies in the substantial cost-effectiveness of our methods, which could be further enhanced by adopting exclusively online data collection tools. The viability of these online methods has been significantly improved by the recent shift towards online education during the COVID-19 pandemic, demonstrating the adaptability and resilience of educational methods. Furthermore, using a validated and structured framework for data collection not only ensures the reliability of the findings provided but also allows for the periodic gathering of data. This aspect is particularly beneficial as it enables the continuous monitoring of evolving adolescent risk patterns within a community, providing a dynamic perspective on public health.

However, our study also encounters several limitations that must be acknowledged. One of the primary constraints is the absence of objective metrics, such as accelerometry for MVPA, and anthropometric or bioelectrical impedance analyses for precise weight status assessment which have been implemented in the ISCOLE study [12]. While our cost-effective and methodologically standardized approach was intended to adapt and streamline the research process, future iterations of this study should consider incorporating these objective measures to enhance the validity of the findings. Notwithstanding, our approach is based on previously documented adequate general accuracy of self-reported BMI in capturing adolescent weight categories [24]. Additionally, our study’s relatively small sample size, though sufficient for local documentation of obesogenic lifestyle practices, may present itself as a further limitation. This is counterbalanced to some extent by the local focus of our study, which aims to identify specific risk factors and inform targeted educational interventions. Under this consideration, the sample size implemented in the current study is of a comparable magnitude with other school-centered investigations [68]. 

Finally, the reliance on self-reported data raises concerns regarding social desirability and recall biases. Social desirability bias may lead participants to provide responses they perceive as socially acceptable or favorable rather than truthful ones, potentially skewing the results towards more positive self-representations or underreporting undesirable behaviors. Recall bias introduces another layer of complexity, as participants might not accurately remember or report past behaviors or conditions. These considerations represent known trade-offs that can affect the validity of research outcomes; which are anticipated when adopting highly cost-effective data collection methodologies. They are, however, integral to the strategic planning of research; especially when balancing between methodological rigor and cost-efficiency, and are inherent to the process of employing economical data collection strategies.

### 4.5. Future Research Directions

The current study represents a natural continuation of our previous research on the potential influence of the school environment on the weight status of children, also carried out through the adaptation and validation of an ISCOLE questionnaire [16]. In a similar effort, the present study aims, first and foremost, to adapt and validate a standardized instrument for data collection and analysis within a specific educational context as a pilot exploratory study. While the sample utilized was relatively small, our initial approach opens the possibility of applying this instrument on samples with statistically significant dimensions. The scientific adaptation and validation of the questionnaires used in this study open important perspectives for extensive research on the school population in Romania. Viewed as direct support for preventive medicine, applying the questionnaires to statistically significant samples for a certain school-age group at the national level, possibly with a certain periodicity to identify trends over time, could serve as an important tool for educational initiatives and may support decision-makers in formulating policies regarding education and public health. 

Furthermore, the data collection model used in the present study allows for immediate implementation at the level of each school, for example, at the beginning of an academic cycle, with the support of school medical staff for the objective collection of necessary information (e.g., BMI). With the transition to an electronic version of the application of the questionnaire for parents, costs would be significantly reduced, and local data collection, followed by national data concatenation, could become relatively simple and fast. 

Moreover, data interpretation tailored to local contexts could lead to specific interventions addressing identified issues. Potential initiatives include launching fitness tracking competitions to reduce sedentary behavior, collaborating with school psychologists to enhance sleep awareness, and organizing inclusive projects to encourage physical education participation among overweight children. Further research efforts could subsequently gauge the efficiency of such interventions.

## 5. Conclusions

In summary, the current research has effectively adapted a series of standardized questionnaires to investigate the lifestyle habits and dietary patterns of Romanian adolescents and their families, providing valuable insights for targeted health education and intervention within a local context. While the results underscore concerns regarding activity levels and insufficient sleep, they also highlight opportunities for creating adaptive health interventions. Despite encountering certain methodological constraints, such as biases inherent to self-reported data and a limited sample size, the study’s approach demonstrates substantial promise for cost-effective and regular data collection. These efforts contribute not only to the immediate understanding of adolescent health behaviors but also to the long-term goal of enhancing health promotion and disease prevention strategies nationally. Further refinement and broader application of the tools developed may aid in shaping future initiatives regarding informed public health policies and educational programs, with the ultimate aspiration of fostering healthier societal conditions.

## Figures and Tables

**Table 1:** Categorical variables related to demographic factors and their relationship to student weight status (percentages of weight status within variable categories).

Variable	Categories	Overweight or Obese		*p*-Value
		No	Yes	
Gender	Female	63 (94%)	4 (6%)	<0.01
	Male	36 (76.6%)	11 (23.4%)	
Living environment (real)	Rural	15 (88.2%)	2 (11.8%)	0.606
	Urban	84 (86.6%)	13 (13.4%)	
Estimated family income (missing = 2)	Below average	5 (100%)	0 (0%)	1
	Average	65 (86.7%)	10 (13.3%)	
	Above average	28 (87.5%)	4 (12.5%)	
Maternal higher education	No (ISCED ≤ 4)	32 (78%)	9 (22%)	0.037
	Yes (ISCED > 4)	67 (91.8%)	6 (8.2%)	
Paternal higher education	No (ISCED ≤ 4)	40 (85.1%)	7 (14.9%)	0.646
	Yes (ISCED > 4)	59 (88.1%)	8 (11.9%)	

ISCED—International Standard Classification of Education.

**Table 2:** Categorical variables related to child and family health and their relationship to student weight status (percentages of weight status within variable categories).

Variable	Categories	Overweight or Obese		*p*-Value
		No	Yes	
Term birth (missing = 1)	Pre-term	7 (87.5%)	1 (12.5%)	0.588
	On term	85 (87.6%)	12 (12.4%)	
	Post-term	6 (75%)	2 (25%)	
Birth weight (missing = 5)	>4000 g	5 (55.6%)	4 (44.4%)	<0.01
	≤4000 g	90 (90%)	10 (10%)	
Maternal age at conception	<30 years	68 (87.2%)	10 (12.8%)	1
	≥30 years	31 (86.1%)	5 (13.9%)	
Paternal age at conception	<30 years	46 (86.8%)	7 (13.2%)	0.988
	≥30 years	53 (86.9%)	8 (13.1%)	
Feeding during first 6 months (missing = 1)	Breastfed (no formula)	60 (89.6%)	7 (10.4%)	0.241
	Formula (no breastfeeding)	7 (70%)	3 (30%)	
	Combined	31 (86.1%)	5 (13.9%)	
Maternal obesity	No	94 (91.3%)	9 (8.7%)	<0.01
	Yes	5 (45.5%)	6 (54.5%)	
Paternal obesity	No	79 (88.8%)	10 (11.2%)	0.314
	Yes	20 (80%)	5 (20%)	

**Table 3:** Numerical variables related to child and family health across student weight categories.

Variable	Weight Status		*p*-Value
	Normal Weight	Overweight or Obese	
	Mean (95% CI) ± StdDev/Median; MIN–MAX (IQR)		
Maternal age (years)	44.62 (43.78–45.46) ± 4.22	44.27 (42.18–46.35) ± 3.77	0.762
	44; 34–53 (6)	43; 37–51 (5)	
Paternal age (years)	47.34 (46.39–48.29) ± 4.76	48 (44.92–51.08) ± 5.57	0.171
	47; 38–66 (6)	47; 8–56 (10)	
Quality of life score	35.78 (34.58–36.95) ± 5.93	37.67 (34.51–40.82) ± 5.69	0.248
	36; 20–50 (8)	38; 30–50 (10)	

95% CI—95% confidence interval for the mean; StdDev—standard deviation; MIN—minimum observed value; MAX—maximum observed value; IQR—interquartile range.

**Table 4:** Categorical variables related to physical activity and sleep and their relationship to student weight status (percentages of weight status within variable categories).

Variable	Categories	Overweight or Obese		*p*-Value
		No	Yes	
Active transport category	1	76 (87.4%)	11 (12.6%)	0.454
	2	10 (76.9%)	3 (23.1%)	
	3	13 (92.9%)	1 (7.1%)	
Weekday sleep duration	<8 h	73 (89%)	9 (11%)	0.355
	≥8 h	26 (81.3%)	6 (18.8%)	
Days participating in PE during school week	0	7 (58.3%)	5 (41.7%)	<0.01
	≥1	92 (90.2%)	10 (9.8%)	
Walking distance from home to public spaces with sports equipment (missing = 30)	>10 min	50 (79.4%)	13 (15.5%)	0.017
	≤10 min	21 (100%)	0 (0%)	

PE—physical education.

**Table 5:** Numerical variables related to physical activity and sedentary time across student weight categories.

Variable	Weight Status		*p*-Value
	Normal Weight	Overweight or Obese	
	Mean (95% CI) ± StdDev/Median; MIN–MAX (IQR)		
Hours spent outside (daily average in a week)	3.38 (3.14–3.61) ± 1.19	3.08 (2.28–3.88) ± 1.44	0.364
	3.21; 0.86–6.14 (1.64)	3.36; 1–6.43 (1.86)	
Average daily non-academic screen time in a week (hours)	2.57 (2.24–2.89) ± 1.62	2.65 (1.88–3.42) ± 1.39	0.75
	2.5; 0–7 (2.28)	2.57; 0.57–4.86 (2.29)	
Average non-academic screen time during weekends (hours)	2.96 (2.6–3.3) ± 1.84	4.1 (3.1–5.1) ± 1.8	0.031
	3; 0–8 (2.5)	4; 1–7 (3)	
Average time spent watching television during weekends (hours)	1.14 (0.91–1.37) ± 1.15	1.9 (1.09–2.71) ± 1.45	0.03
	0.5; 0–5 (2)	2; 0–5 (2.5)	
Average time spent using the computer during weekends (hours)	1.83 (1.51–2.13) ± 1.51	2.2 (1.29–3.11) ± 1.65	0.422
	2; 0–5 (2.5)	2; 0–5 (2.5)	
Number of days a week of at least 60 min MVPA	3.34 (2.94–3.74) ± 2.01	3.6 (2.67–4.53) ± 1.68	0.568
	3; 0–7 (3)	4; 0–7 (3)	
Intrinsic Motivation for PA Score	19.32 (18.65–20) ± 3.4	19.73 (18.13–21.33) ± 2.89	0.923
	20; 11–25 (4)	21; 14–24 (5)	
Self-Efficacy for PA Score	27.39 (26.13–28.66) ± 6.34	28.47 (25.23–31.71) ± 5.85	0.382
	28; 10–40 (9)	29; 18–40 (8)	

95% CI—95% confidence interval for the mean; StdDev—standard deviation; MIN—minimum observed value; MAX—maximum observed value; IQR—interquartile range; MVPA–moderate-to-vigorous physical activity; PA–physical activity.

**Table 6:** Weekly portions obtained from FFQ and in front of screens—totals and components with significant correlation to weight status, and Emotion-induced eating score across weight categories.

Variable	Weight Status		*p*-Value
	Normal Weight	Overweight or Obese	
	Mean (95%CI) ± StdDev/Median; MIN–MAX (IQR)		
Total weekly unhealthy food portions	16.22 (13.62–18.82) ± 13.04	12.97 (8.51–17.42) ± 8.05	0.644
	13; 0–100 (12.5)	12; 0–27.5 (12.5)	
Total weekly healthy food portions)	30.97 (27.37–34.58) ± 18.06	29.93 (21.89–37.98) ± 14.53	0.957
	27; 0–100 (24)	27.5; 11–70.5 (14)	
Weekly portions of chips	1.09 (0.76–1.42) ± 1.66	0.2 (0–0.43) ± 0.41	0.026
	1; 0–10 (1)	0; 0–1 (<0.01)	
Weekly portions of fruit juice	2.71 (2.2–3.22) ± 2.56	1.23 (0.32–2.15) ± 1.66	0.049
	1; 0–10 (4.5)	1; 0–5.5 (3)	
Total weekly food portions consumed in front of screens	9.51 (7.88–11.14) ± 8.17	8.87 (3.95–13.78) ± 8.89	0.930
	7; 0–41.5 (11)	7.5; 0–37 (9)	
Weekly portions of chips consumed in front of screens	0.91 (0.68–1.15) ± 1.17	0.37 (0–0.8) ± 0.79	0.032
	0.5; 0–5.5 (1)	0; 0–3 (0.5)	
Emotion-induced eating score	11.31 (10.73–11.89) ± 2.9	12.4 (10.85–13.95) ± 2.8	0.387
	11; 7–18 (5)	11; 9–20 (4)	

95%CI—95% confidence interval for the mean; StdDev—standard deviation; MIN—minimum observed value; MAX—maximum observed value; IQR—interquartile range.

**Table 7:** Variables derived from the CFQ across weight categories.

Variable	Weight Status		*p*-Value
	Normal Weight	Overweight or Obese	
	Mean (95%CI) ± StdDev/Median; MIN–MAX (IQR)		
Monitoring high-fat foods	3.55 (3.32–3.79) ± 1.15	4.27 (3.78–4.76) ± 0.88	0.019
	4; 1–5 (1)	4; 2–5 (1)	
Perceived parent weight in the present	2.24 (2.15–2.34) ± 0.48	2.67 (2.32–3.01) ± 0.62	<0.01
	2; 1–4 (<0.01)	3; 2–4 (1)	
Perceived child weight during first year of life	1.93 (1.86–1.99) ± 0.336	2.13 (1.85–2.42) ± 0.516	0.049
	2; 1–3 (<0.01)	2; 1–3 (<0.01)	
Perceived child weight during age 2 to 3	1.95 (1.88–2.01) ± 0.31	2.2 (1.97–2.43) ± 0.41	<0.01
	2; 1–3 (<0.01)	2; 2–3 (<0.01)	
Perceived child weight during preschool	1.86 (1.79–1.93) ± 0.35	2.2 (1.97–2.43) ± 0.41	<0.01
	2; 1–2 (<0.01)	2; 2–3 (<0.01)	
Perceived child weight during primary classes	1.93 (1.86–1.99) ± 0.34	2.27 (2.01–2.52) ± 0.46	<0.01
	2; 1–3 (<0.01)	2; 2–3 (1)	
Perceived child weight during Middle School	2.01 (1.93–2.09) ± 0.37	2.47 (2.11–2.82) ± 0.64	<0.01
	2; 1–3 (<0.01)	2; 2–4 (1)	
Perceived child weight during High School	1.94 (1.89–1.99) ± 0.25	2.53 (2.18–2.89) ± 0.64	<0.01
	2; 1–2 (<0.01)	2; 2–4 (1)	
Concern regarding child weight	1.78 (1.57–1.98) ± 1	3 (2.25–3.75) ± 1.36	<0.01
	1; 1–5 (2)	3; 1–5 (2)	
Restriction of sweets	3.88 (3.66–4.11) ± 1.1	4.47 (4.11–4.82) ± 0.64	0.041
	4; 1–5 (1)	5; 3–5 (1)	

95%CI—95% confidence interval for the mean; StdDev—standard deviation; MIN—minimum observed value; MAX—maximum observed value; IQR—interquartile range.

**Table 8:** Questionnaire internal consistency.

Questionnaire	Question Interval	No. of Items after Adaptation	Cronbach’s Alpha (Adapted Items)
ISCOLE-LDQ	13–20 (Self-Efficacy for PA)	8	0.849
	21–25 (Intrinsic Motivation for PA)	5 (RC: 21, 23)	0.52
	
	33 (23-FFQ)	23	0.877
	34 (Screen FFQ)	9	0.753
	39–45 (Emotional eating)	7	0.723
	46–56 (Kidscreen-10)	11 (RC according to official instructions)	0.798
CFQ	1–3 (Perceived Responsibility)	3	0.703
	4, 5, 7		
	(Perceived Parent Weight)	3	0.438
	8–13 (Perceived Child Weight)		
		6	0.734
	14, 16 (Concern)	2	0.671
	17–23 + new question (Restriction)	8	0.726
	25–28 (Pressure to Eat)	4	0.677
	29–31 (Monitor)	3	0.869
ISCOLE-NHEQ	Section D, 1–17	16	0.7
	(Foods in the Home)		
	Section E, 1–5	6	0.585
	(Where you Shop)		
	Section G, 1–5 (Food Shopping)	5	0.275
	Section H, 1–5 (Child’s Play Equipment)	8	0.597
	Section J, 1–13 (Child’s Places for Physical Activity)	12	0.729
	Section L, 1–13	9	0.795
	(Distance to Locations)		
	Section M, 1–4	4	0.488
	(Family)		

ISCOLE-LDQ—ISCOLE Lifestyle and Diet Questionnaire; FFQ—Food Frequency Questionnaire; CFQ—Child Feeding Questionnaire; ISCOLE-NHEQ—ISCOLE Neighbourhood and Home Environment Questionnaire; PA—physical activity, RC—reverse-coded.

**Table 9:** Summary of statistically significant correlations with weight status.

Investigated Variables	Weight Status Correlation	Interpretation/Previous Findings
Gender	Higher values in males	Previously described by Shah et al. [54]
Maternal higher education	Inverse	Previously described variable correlation associated with country socio-economical status (Katzmarzyk et al.) [13]
Maternal obesity	Positive	Similar results by Mannino et al. [55]
Birth weight	Positive	Similar results by Qiao et al. [29]
Participation in PE classes	Inverse	Similar results by Cawley et al. [56]
Walking distance from home to public spaces with sports equipment	Positive	May offer future research directions regarding the impact of publicly available physical activity resources
Non-academic screen time during weekends (mostly due to television watching)	Positive	Integral part of previously described effects of sedentary time on childhood weight balance [13]
Consumption of potato chips	Inverse	Possibly due to social desirability bias
Parental monitoring of high-fat foods	Positive	Similar findings by Birch et al. [10], and Kaur et al. [11]
Parental concern regarding child weight		
Parental restriction of sweets		
Parental self-perceived weight (present)		
Parental perceived weight of child (all periods)		

## Data Availability

The data presented in this study are available upon reasonable request from the corresponding author.

## Supplementary Materials

The following supporting information can be downloaded at: https://www.mdpi.com/article/10.3390/nu16101532/s1, Table S1: Original ISCOLE, CFQ questionnaires and COSI questions, forward and backward translations or adaptations; Table S2: Changelogs for the modifications made to the questionnaires utilized; Table S3: Student average marks at the end of the year across various categories; Figure S1: Parental perception of child weight category; Figure S2: Parental self-perception of weight category; Scan S1: Backward translation 1; Scan S2: Backward translation 2.
