# Supplementary material for: Assessing Lifestyle Patterns and Their Influence on Weight Status in Students from a High School in Sibiu, Romania: An Adaptation of ISCOLE Questionnaires and the Child Feeding Questionnaire
Source: Nutrients. 2024 May 20;16(10):1532. doi: 10.3390/nu16101532 (PMC11123863; doi:10.3390/nu16101532)
Supplement: Supplementary file 1 [file nutrients-16-01532-s001.zip › Scan S1. Backward translation 1.pdf]

Diet and lifestyle

Please read each question carefully. What's the answer that comes to your mind first? Mark at each question the answer that suits you best.

**To remember:**

- This is not a test so there are no wrong answers.
- You don't have to show anyone your answers.
- No one who knows you will see your answers.

1. Please write your first and last name in the next space:
2. How old are you? (please use only numbers to answer)
3. Last week **on a school day** how many hours did you watch TV?

   I didn't watch TV on school days / less than 1 hour /1 hour/ 2 hours/ 3 hours / 4 hours / 5 or more hours
4. Last week **on a school day**, how many hours did you play video or computer games or did you use your computer for activities other than those required for school?

   During school days I did not play video or computer games and did not use the computer for activities other than those required for school / less than 1 hour / 1 hour / 2 hours / 3 hours / 4 hours / 5 or more hours
5. Last week **on a school day**, about how many hours did you spend outside **before** going to school?

   Less than 1 hour / 1 hour / 2 hours / 3 hours / 4 hours / 5 or more hours
6. Last week **on a school day**, about how many hours you did you spend outside **after** being to school?

   Less than 1 hour / 1 hour / 2 hours / 3 hours / 4 hours / 5 or more hours
7. Last week on a weekend day, how many hours did you watch TV for?

   I didn't watch TV on weekends / less than 1 hour / 1 hour / 2 hours / 3 hours / 4 hours / 5 or more hours
8. Last week **on a weekend day**, how many hours have you played video or computer games or used your computer for activities other than those required for school?

   On weekends I did not play video or computer games and did not use the computer for activities other than those required for school / less than 1 hour / 1 hour / 2 hours / 3 hours / 4 hours / 5 or more hours
9. Last week **on a weekend day** how many hours did you spend outside? Less

   than 1 hour / 1 hour / 2 hours / 3 hours / 4 hours / 5 or more hours
10. Last week at school, how many days did you go to physical education and sports

    classes? 0 / 1 Day / 2 days / 3 days / 4 days / 5 days

*The undersigned, Matei Rodica, authorised interpret and translator for the foreign languages English-French-Spanish based on the authorisation no. 29028 from 2010, issued by the Ministry of Justice from Romania, certify the accuracy of the translation made from Romanian into English that the text shown to me was translated in excerpt without omissions and that by the translation the content and the meaning of the document was not modified.*

MATEI RODICA
Engleză, Franceză Spaniolă

11. How do you travel most of your way from home to school?

Walk / by bicycle, scooter (not electric), roller or skateboard / by bus, trolleybus, tram or other means of public transport / by car, motorcycle, moped, electric scooter / other options

12. If you answered" other options " to the previous question, please explain:

13. How long does it take to get from home to school?

Less than 5 minutes / 5-15 minutes / 16-30 minutes / between 30 minutes and an hour / over 1 hour

14. Which of the following activities have you participated in in the last 12 months?

Team sports games / dance, martial arts / art, music / none of the above

15. In the last 7 days, how many days have you been physically active for at least 60 Minutes a day? (activities that make your heart beat faster and make you breathe faster than usual)

0 / 1 Day / 2 days / 3 days / 4 days / 5 days / 6 days / 7 days

**For each of the following 8 statements, please select a response between 1 and 5:**

1 = if you completely disagree with the statement 2 = if you

disagree with the statement

3 = if you are undecided about the claim 4 = if you agree

with the statement

5 = if you fully agree with the statement

16. I choose to be physically active in my free time most days.

17. I can ask my parents or other adults to do physical activities with me.

18. I Prefer to be physically active in my free time most days, even though instead of physical activities I could watch TV or play video games.

19. I Prefer to be physically active in my free time most days, even if it is very hot or very cold outside.

20. I can ask my best friend to do physical activities with me in my spare time most days.

21. I Prefer to be physically active in my free time most days, even if I have to stay at home

22. I have the coordination and motivation to be physically active in my free time most days.

23. I Prefer to be physically active in my free time most days no matter how busy my day has been.

*The undersigned, Matei Rodica, authorised interpret and translator for the foreign languages English-French-Spanish based on the authorisation no. 29028 from 2010, issued by the Ministry of Justice from Romania, certify the accuracy of the translation made from Romanian into English that the text shown to me was translated in excerpt without omissions and that by the translation the content and the meaning of the document was not modified.*

CIF 27195290 MATEI RODICA Engleză, Franceză Spaniolă

**There are many reasons why people participate in physical activities. For each of the following 5 statements, please select a response between 1 and 5:**

1 = if the statement is never true to you 2 = if the statement is rarely true to you

3 = if the statement is sometimes true for you 4 = if the statement is often true for you

5 = if the statement is always true for you

24. I take part in exercise because other people tell me I should do it.
25. It is important for me to exercise regularly.
26. I don't see why I should bother exercising.
27. I have the feeling of failure when I have not exercised for a period of time.
28. For me exercise is a pleasant activity

**For the next 4 questions, write down the time and minute that best correspond to your situation. Please use the 24-hour format (see example). For example: if I go to bed at 10 in the evening, then I write 22:00**

29. Last week, what time did you turn off the lights and go to bed routinely **on school days**?
30. Last week, what time did you typically wake up **on school days**?
31. Last week, what time did you turn off the lights and go to bed **on weekends**?
32. Last week, what time did you typically wake up **on weekends**?
33. How do you assess how well you slept last week?

    Very good / pretty good / medium / pretty bad / very bad
34. How do you appreciate how much you slept last week? (how long have you slept) very much / much / enough / Little / very little
35. Do you have a TV in the room you sleep in? Yes/No
36. Do you have a computer in the room where you sleep? Yes/No
37. How many days a week do you usually eat

    Fruits / vegetables / sweets, candies, chocolates / sugar-containing juices / cakes, pastry shops, Donuts / diet juices (without sugar) / potato chips / french fries / green vegetables (broccoli, spinach, etc.) / orange vegetables (carrot, pumpkin, sweet potato, etc.) / fruit juice / skim milk (1-2% fat) / whole milk / cheese / other dairy (yogurt, pudding, etc.) / whole or whole grain bread (oats, muesli, etc.) / alternatives to meat (beans, lentils, tofu peanut butter, etc.) / energy drinks (Red Bull, Monster, hell, etc.) / sports drinks (eg. Gatorade) / fish / ice cream / fried food / Fast food (pizza, hamburgers, etc.)

*The undersigned, Matei Rodica, authorised interpret and translator for the foreign languages English-French-Spanish based on the authorisation no. 29028 from 2010, issued by the Ministry of Justice from Romania, certify the accuracy of the translation made from Romanian into English that the text shown to me was translated in excerpt without omissions and that by the translation the content and the meaning of the document was not modified.*

MATEI RODICA
Engleză, Franceză
Spaniolă

Never / less than once / once / 2-4 days / 5-6 days / once a day, every day / several times a day, every day

38. How many days a week do you usually eat the following while looking at a screen (TV or computer or phone)?

    Potato chips / salted peanuts / sunflower seeds / fried foods (french fries, chicken wings, etc.) / cakes, biscuits, chocolate / ice cream / Fast food (pizza, hamburgers, etc.) / fruit or vegetables / nuts or peanuts not roasted and unsalted

    Never / less than once / once / 2-4 days / 5-6 days / once a day, every day / several times a day, every day

39. How often do you eat breakfast **on weekdays**, except weekends? (more than a glass of milk or a glass of fruit juice)

    Never/ one day a Week / 2 days a Week / 3 days a Week / 4 days a Week / 5 days a week

40. How often do you eat breakfast **on weekends**? (more than a glass of milk or a glass of fruit juice)

    Usually never / usually Saturday or Sunday / usually also Saturday and Sunday

41. Does your school offer lunch for students?

    Yes / No

42. Last week, how many days did you eat lunch at school? Never /

    one day / 2 days / 3 days / 4 days / 5 days

43. Last week, at how many meals in total (breakfast, lunch or dinner) did you consume products that were not prepared in the home (for example, at the restaurant, fast food, etc.)?

44. How well do the following statements suit you?

    I eat more when I'm worried / I eat when I'm angry / when I do something right, I reward myself with food / I eat more when I'm sad / I eat more when I'm happy / I eat more when I'm bored / I eat between meals even though I'm not hungry

    Never or almost never / sometimes / usually or always

45. If you think about last week...

    Did you feel well and fit? / Did you feel full of energy? / Did you feel sad? / Did you feel lonely? / Have you had enough time for yourself? / Did you do the things you wanted to do in your free time? / Did your parents treat you well? / Did you have fun with your friends? / Did you do well at school? / Did you manage to pay attention at school?

    Not At All / Little / Moderate / Very / Extreme

46. In general, how do you assess that your health is?

    Excellent / very good / good / acceptable / bad / very bad

*The undersigned, Matei Rodica, authorised interpret and translator for the foreign languages English-French-Spanish based on the authorisation no. 29028 from 2010, issued by the Ministry of Justice from Romania, certify the accuracy of the translation made from Romanian into English that the text shown to me was translated in excerpt without omissions and that by the translation the content and the meaning of the document was not modified.*

CIF 27195290 MATEI RODICA Engleză, Franceză Spaniolă

Questionnaire for parents / guardians - Obesity study
Please read each question carefully.
Mark on each question the answer that suits you best.

**To remember:**

- This is not a test, so there are no wrong answers.
- The data obtained are confidential

1. Please fill in your first and last name

2. Please fill in a contact number

*Your telephone number will only be used to contact them in connection with the collection of data required for this study.*

3. What is your relationship with the child included in the study?

Mother / stepmother / grandmother / father / stepfather /

grandfather / other variant

4. What is the name and surname of the child in your care who was included in this study? If you have several children included in the study, please fill out a questionnaire for each of them.

5. What environment is the child's home

in? Urban / Rural

6. What is the date of birth of the child?

7. What is the gender of the child?

Male / Female

8. In which country was the child born?

9. How many brothers and sisters does the child have in your care? (check two boxes) no

sister/one sister/two sisters/three sisters/four sisters/five sisters/more than five sisters No brother /

one brother / two brothers / Three Brothers / Four Brothers / five brothers/more than five brothers

10. If the child in your care has Brothers or sisters, please write for each of them the age, weight and height in the following format: Brother 1-Age ( Years) - Weight (kg) - height (cm)

11. What school is your child enrolled in?

12. What class is the child in?

13. What was the weight in grams at birth of the child?

14. Was the baby born at term?

Yes – no-premature / no - pregnancy

15. If you answered No to the previous question, please specify the duration of pregnancy in weeks.

16. Did the mother of the child develop gestational diabetes during

pregnancy? Yes/No

*The undersigned, Matei Rodica, authorised interpret and translator for the foreign languages English-French-Spanish based on the authorisation no. 29028 from 2010, issued by the Ministry of Justice from Romania, certify the accuracy of the translation made from Romanian into English that the text shown to me was translated in excerpt without omissions and that by the translation the content and the meaning of the document was not modified.*

CIF 27195290 MATEI RODICA Engleză, Franceză Spaniolă

17. Was the baby breastfed? Yes/No

18. If you answered yes to the previous question, at what age (in months) did the baby stop breastfeeding?

19. Was the baby formula fed?

Yes/No

20. If to the previous question the answer was yes, at what age in months did you start feeding the baby with Formula?

21. If you fed your baby formula, at what age in months did you stop formula feeding?

22. What is the marital status of the child's parents? Married /

divorced or separated/never married/widowed

23. How many people live with the child in your care?

Biological mother / stepmother/biological father/stepfather/brothers and sisters/grandparents/friends/other relatives / other people

24. What category do you think your monthly family income currently falls into?

Below average/average/above average

25. How many motorized vehicles (cars, motorcycles, scooters, etc.) do you have in your possession or at your disposal?

26. How many TVs are available at the home of the child in your care?

27. What is the highest successfully graduated level of education of the mother of the

child? No Graduate School / Primary Education (grades 1-4) / secondary education

(grades 5-8)

High school / vocational school / post-secondary school / university education / Master's / doctoral / postdoctoral studies

28. How many hours does the mother of the child work outside the home?

0 (housewife) / less than 20 hours/week / 20 hours per week (part time) / 40 hours per week (full time) / over 40 hours per week

29. What is the highest successfully graduated level of education of the child's father?

High school / vocational school / post-secondary school / university education / Master's / doctoral / postdoctoral studies

30. How many hours does the child's father work outside the home?

0 (domestic) / less than 20 hours / week / 20 hours per week (part time) / 40 hours per week (full time) / over 40 hours per week

31. What is the current age of the mother of the child?

32. What is the current weight (in kilograms) of the mother of the child?

33. What is the current height (in centimeters) of the mother of the child?

34. What is the current age of the child's father?

*The undersigned, Matei Rodica, authorised interpret and translator for the foreign languages English-French-Spanish based on the authorisation no. 29028 from 2010, issued by the Ministry of Justice from Romania, certify the accuracy of the translation made from Romanian into English that the text shown to me was translated in excerpt without omissions and that by the translation the content and the meaning of the document was not modified.*

MATEI RODICA
Engleză, Franceză

35. What is the current weight (in kilograms) of the child's father?

36. What is the current height (in centimeters) of the child's father?

37. When your child is at home, how often are you responsible for feeding them?

1-Never; 2-rarely; 3-on half the occasions; 4-often; 5-Always

38. How often are you responsible for deciding **how large portions** your child eats?

1-Never; 2-rarely; 3-on half the occasions; 4-often; 5-Always

39. How often are you responsible for deciding whether the type of food your child eats is appropriate?

1-Never; 2-rarely; 3-on half the occasions; 4-often; 5-Always

40. How frequently do you monitor the consumption of sweets (chocolate, ice cream, candy, pastries) or sugar-sweetened juices of the child in your care?

1-Never; 2-rarely; 3-on half the occasions; 4-often; 5-Always

41. How frequently do you monitor the consumption of snack foods (potato chips, puffs, etc.) of the child in your care?

1-Never; 2-rarely; 3-on half the occasions; 4-often; 5-Always

42. How frequently do you monitor the consumption of high-fat foods of the child in your care?

1-Never; 2-rarely; 3-on half the occasions; 4-often; 5-Always

43. How do you perceive **your** weight during the following periods:

Underweight / Normoweight / Overweight / Obesity / Extreme obesity

Childhood / Adolescence / Present

44. How do you perceive the weight of the **child** during the following periods:

Underweight / Normopondere / overweight / obesity / extreme obesity

First year of life / 2-3 years / preschool / primary classes / Middle School / High School

45. How worried Are you about the weight of the child in your care?

NOT AT ALL WORRIED (1 ) - VERY WORRIED (5)

46. How worried Are you that your child may need to follow a diet to achieve and maintain a normal weight?

NOT AT ALL WORRIED (1 ) - VERY WORRIED (5)

For each of the following 11 statements, please select an answer between 1 and 5:

1 = if you totally disagree with that statement
2 = if you disagree with that statement
3 = if you are undecided about the claim
4 = if you agree with that statement
5 = if you totally agree with that statement

*The undersigned, Matei Rodica, authorised interpret and translator for the foreign languages English-French-Spanish based on the authorisation no. 29028 from 2010, issued by the Ministry of Justice from Romania, certify the accuracy of the translation made from Romanian into English that the text shown to me was translated in excerpt without omissions and that by the translation the content and the meaning of the document was not modified.*

MATEI RODICA Engleză, Franceză Spaniolă

47. I have to make sure that the child in my care does not consume too many sweets (chocolate, ice cream, candy, pastries) or sugary juices.

48. I have to make sure that the child in my care does not consume too many high-fat foods.

49. I must make sure that the child in my care does not consume too much of his/her favorite foods, whatever they may be.

50. I intentionally keep certain foods hidden or out of the access of the child in my care.

51. If I did not take care of what the child in my care eats, he would consume too many unhealthy foods.

52. The child in my care must eat everything from the plate.

53. I need to be careful that the child eats enough at the table.

54. I try to convince the child to eat when it's mealtime, even if he says he's not hungry.

55. If I did not guide the child's nutrition, he would eat much less than he needs.

56. If I did not guide the child's nutrition, he would eat much more than he needs.

57. I offer rewards in the form of sweets or sugar-sweetened juices to the child in my care for good behavior.

1-Never 2-Rarely 3 - Sometimes 4-Frequently 5-Always

58. I offer rewards in the form of favorite foods to the child in my care for good behavior.

1-Never 2-Rarely 3 - Sometimes 4-Frequently 5-Always

59. How frequently are the following types of food found in the home of the child enrolled in the study?

Chocolate / other sweets / raw fruits / Dried fruits / cakes, cakes, pastries / potato Chips, pretzels, puffs / unroasted and unsalted nuts and hazelnuts / roasted and salted nuts and hazelnuts / raw vegetables / 100% fruit juices / sugar-sweetened juices / sugar-free juices / whole milk / skimmed milk / sugar cereals / unsweetened cereals

Never / Rarely / Sometimes / Often / Always

60. How frequently do you shop for groceries from the following types of stores?

Hypermarkets (Carrefour, Kaufland, Auchan, etc.) / neighborhood supermarkets / grocery

market / local producers / other

Never / Rarely / Sometimes / Often / Always

61. In the place where you most frequently buy food:

Healthy dishes are too expensive / there is a rich variety of fresh fruits and vegetables / there is a rich variety of healthy dishes / fruits and vegetables

*The undersigned, Matei Rodica, authorised interpret and translator for the foreign languages English-French-Spanish based on the authorisation no. 29028 from 2010, issued by the Ministry of Justice from Romania, certify the accuracy of the translation made from Romanian into English that the text shown to me was translated in excerpt without omissions and that by the translation the content and the meaning of the document was not modified.*

MATEI RODICA
Engleză, Franceză Spaniolă
CIF 27195290

fresh vegetables are in poor condition / fresh fruits and vegetables are too expensive

Total disagreement / disagreement / indifferent / Agreement / total agreement

62. Please mark which of the following electronics are in your child's bedroom TV / computer / laptop / video game system (Playstation, Xbox, Nintendo, etc.) yes / no

63. Please mark which of the following **electronics is available to or in the child's possession**:

Mobile phone / portable gaming system /(Game-Boy, PlayStation Portable, etc.) / music playback system (mp3-player, ipod, etc.) / electronic tablet

YES/NO

64. Please mark how frequently the child in your care has used the following objects/facilities during the last year

Bicycle / basketball basket / jumping rope / active video games (eg. dance, movement) / sports equipment (football or basketball, tennis rackets, etc.) / Rollers, skateboard, scooter (not electric) / swimming pool / fixed play equipment (slides, swings, ping-pong table, etc.)

Not applicable/not available / has available but has not used at all / once a month or less / once every two weeks / Weekly or more often

65. Please mark how frequently your child has had physical activity at the following locations during the past year

Home / in the yard / home to a neighbor or friend / on a street near the home / gym or indoor recreation complexes / at the strand / hiking trails / playgrounds (basketball, football, tennis, etc.) / small neighborhood parks / large public parks / School (extracurricular) / other public spaces

Never / once a month or less / once every two weeks / once a week / 2-3 times a Week / 4 times a week or more often

66. Please mark how long it would take approximately to walk to the following facilities at the home of the child in your care:

Small neighborhood store / Supermarket / fast food Restaurant / gym / swimming pool / playgrounds (basketball, tennis, football, etc.) / small neighborhood parks / large public parks / public spaces with sports equipment

1-5 minutes / 6-10 minutes / 11-20 minutes / 21-30 minutes / over 30 minutes / I don't know

67. During a typical week, how frequently you or another adult in the child's family

Watch the child while practicing physical activities / encourage the child to practice physical activities / Transport the child to a place where they are doing physical activity / practice a physical activity with the child

Never / 1-2 days a week / 3-4 days a week / 5-6 days a week / Daily

*The undersigned, Matei Rodica, authorised interpret and translator for the foreign languages English-French-Spanish based on the authorisation no. 29028 from 2010, issued by the Ministry of Justice from Romania, certify the accuracy of the translation made from Romanian into English that the text shown to me was translated in excerpt without omissions and that by the translation the content and the meaning of the document was not modified.*

CIF 27195290 MATEI RODICA Engleză, Franceză Spaniolă

68. Please mark if the child's **mother** suffers or has suffered from any of these conditions, or if she has ever been treated for any of them:

Hypertension (high blood pressure) / Diabetes mellitus / Obesity / Ischaemic heart disease / Myocardial infarction / Stroke / Chronic obliterating arteriopathy of the lower limbs (narrowing of the arteries in the legs) / Heart failure / Hypercholesterolemia (high blood cholesterol) / Hypertriglyceridemia (high triglycerides in the blood)

69. Please mark if the child's **father** suffers or has suffered from any of these conditions, or if he has ever been treated for any of them:

Hypertension (high blood pressure) / Diabetes mellitus / Obesity / Ischaemic heart disease / Myocardial infarction / Stroke / Chronic obliterating arteriopathy of the lower limbs (narrowing of the arteries in the legs) / Heart failure / Hypercholesterolemia (high blood cholesterol) / Hypertriglyceridemia (high triglycerides in the blood)

*The undersigned, Matei Rodica, authorised interpret and translator for the foreign languages English-French-Spanish based on the authorisation no. 29028 from 2010, issued by the Ministry of Justice from Romania, certify the accuracy of the translation made from Romanian into English that the text shown to me was translated in excerpt without omissions and that by the translation the content and the meaning of the document was not modified.*

MATEI RODICA
Engleză, Franceză
