# Supplementary material for: Assessing Lifestyle Patterns and Their Influence on Weight Status in Students from a High School in Sibiu, Romania: An Adaptation of ISCOLE Questionnaires and the Child Feeding Questionnaire
Source: Nutrients. 2024 May 20;16(10):1532. doi: 10.3390/nu16101532 (PMC11123863; doi:10.3390/nu16101532)
Supplement: Supplementary file 1 [file nutrients-16-01532-s001.zip › Supplementary Materials.pdf]

Table S1 - *Original ISCOLE, CFQ questionnaires and COSI questions, forward and backward translations or adaptations*

	Question, title or description
Original	**ISCOLE Diet and Lifestyle Questionnaire**
Adapted	Chestionar dietă și stil de viață
Backward	Diet and lifestyle questionnaire
Original	Please read every question carefully. What answer comes to your mind first?<br>Choose the box that fits your answer best and fill it in.<br><br>Remember: This is not a test so there are no wrong answers. It is important that you answer all the questions and that we can see your marks clearly.<br><br>You do not have to show your answers to anybody. Also, nobody who knows you will look at your questionnaire once you have finished it.
Adapted	Te rugăm să citești fiecare întrebare cu atenție. Care este răspunsul care îți vine în minte prima dată? Marchează la fiecare întrebare răspunsul care se potrivește cel mai bine.<br>De ținut minte:<br>Acesta nu este un test așa că nu există răspunsuri greșite.<br>Nu trebuie să arăți nimănui răspunsurile tale.<br>Nimeni dintre cei care te cunosc nu vor vedea răspunsurile tale.
Backward	Please read each question carefully. What's the answer that comes to your mind first? Mark each question with the answer that suits you best.<br>Remember<br>- This is not a test so there are no wrong answers.<br>- You don't have to share your answers with anyone.<br>- No one who knows you will see your answers.
Original	(none)
Adapted	Te rugăm scrie numele și prenumele tău în spațiul următor
Backward	Please write your first and last name in the next space:
Original	(none)
Adapted	Câți ani ai? (te rugăm să folosești numai numere pentru răspuns)
Backward	How old are you? (please use only numbers to answer)
Original	1. On a <u>school day</u>, how many hours did you watch TV?<br>☐ I did not watch TV on <u>school</u> days ☐ < 1 hour ☐ 1 hour ☐ 2 hours ☐ 3 hours ☐ 4 hours ☐ 5 or more hours
Forward	*Săptămâna trecută* într-o <u>zi de școală</u>, cam câte ore te-ai uitat la televizor?<br>*Nu m-am uitat la televizor în zilele de școală/Mai puțin de 1 oră/1 oră/2 ore/3 ore/4 ore/5 sau mai multe ore*
Backward	*Last week* on a <u>school day</u> how many hours did you watch TV?<br>*I didn't watch TV on school days / less than 1 hour /1 hour/ 2 hours/ 3 hours / 4 hours / 5 or more hours*
Original	2. On a <u>school day</u>, how many hours did you play video or computer games or use a computer for something that was <u>not</u> school work?<br>☐ I did not play video/computer games or use a computer other than for school work on <u>school</u> days ☐ < 1 hour ☐ 1 hour ☐ 2 hours ☐ 3 hours ☐ 4 hours ☐ 5 or more hours
Forward	*Săptămâna trecută* într-o <u>zi de școală</u>, cam câte ore te-ai jucat jocuri video sau pe calculator sau ai folosit calculatorul pentru <u>alte</u> activități decât cele necesare pentru școală?<br>*În zilele de școală nu m-am jucat jocuri video sau pe calculator și nu am folosit calculatorul pentru alte activități decât cele necesare pentru școală/Mai puțin de 1 oră/1 oră/2 ore/3 ore/4 ore/5 sau mai multe ore*
Backward	*Last week* on a <u>school day</u>, how many hours did you play video games or use your computer for activities other than those required for schoolwork?

	During school days I did not play video or computer games and did not use the computer for activities other than those required for school / less than 1 hour / 1 hour / 2 hours / 3 hours / 4 hours / 5 or more hours
Original	3. On a school day how much time did you spend outside **before** school?<br>☐ < 1 hour ☐ 1 hour ☐ 2 hours ☐ 3 hours ☐ 4 hours ☐ 5 or more hours
Forward	*Săptămâna trecută* într-o zi de școală, cam câte ore ai petrecut afară ÎNAINTE de a merge la școală?<br>*Mai puțin de 1 oră/1 oră/2 ore/3 ore/4 ore/5 sau mai multe ore*
Backward	*Last week* on a school day, about how many hours did you spend outside BEFORE going to school?<br>*Less than 1 hour / 1 hour / 2 hours / 3 hours / 4 hours / 5 or more hours*
Original	4. On a school day how much time did you spend outside **after** school before bedtime?<br>☐ < 1 hour ☐ 1 hour ☐ 2 hours ☐ 3 hours ☐ 4 hours ☐ 5 or more hours
Forward	*Săptămâna trecută* într-o zi de școală, cam câte ore ai petrecut afară DUPĂ ce ai fost la școală?<br>*Mai puțin de 1 oră/1 oră/2 ore/3 ore/4 ore/5 sau mai multe ore*
Backward	*Last week* on a school day, about how many hours you did you spend outside AFTER school?<br>*Less than 1 hour / 1 hour / 2 hours / 3 hours / 4 hours / 5 or more hours*
Original	5. On a weekend day, how many hours did you watch TV?<br>☐ I did not watch TV on weekend days ☐ < 1 hour ☐ 1 hour ☐ 2 hours ☐ 3 hours ☐ 4 hours ☐ 5 or more hours
Forward	*Săptămâna trecută* într-o zi de weekend, cam câte ore te-ai uitat la televizor?<br>*Nu m-am uitat la televizor în zilele de weekend/Mai puțin de 1 oră/1 oră/2 ore/3 ore/4 ore/5 sau mai multe ore*
Backward	*Last week* on a weekend day, how many hours did you watch TV?<br>*I didn't watch TV on weekends / less than 1 hour / 1 hour / 2 hours / 3 hours / 4 hours / 5 or more hours*
Original	6. On a weekend day, how many hours did you play video or computer games or use a computer for something that was not school work?<br>☐ I did not play video/computer games or use a computer other than for school work on the weekend ☐ < 1 hour ☐ 1 hour ☐ 2 hours ☐ 3 hours ☐ 4 hours ☐ 5 or more hours
Forward	*Săptămâna trecută* într-o zi de weekend, cam câte ore te-ai jucat jocuri video sau pe calculator sau ai folosit calculatorul pentru alte activități decât cele necesare pentru școală?<br>*În zilele de weekend nu m-am jucat jocuri video sau pe calculator și nu am folosit calculatorul pentru alte activități decât cele necesare pentru școală/Mai puțin de 1 oră/1 oră/2 ore/3 ore/4 ore/5 sau mai multe ore*
Backward	Last week on a weekend day, how many hours did you play video games or use your computer for activities other than those required for school?<br>*On weekends I did not play video or computer games and did not use the computer for activities other than those required for school / less than 1 hour / 1 hour / 2 hours / 3 hours / 4 hours / 5 or more hours*
Original	7. On a weekend day, how much time did you spend outside?<br>☐ < 1 hour ☐ 1 hour ☐ 2 hours ☐ 3 hours ☐ 4 hours ☐ 5 or more hours
Forward	*Săptămâna trecută* într-o zi de weekend, cam câte ore ai petrecut afară?<br>*Mai puțin de 1 oră/1 oră/2 ore/3 ore/4 ore/5 sau mai multe ore*
Backward	Last week on a weekend day how many hours did you spend outside?<br>*Less than 1 hour / 1 hour / 2 hours / 3 hours / 4 hours / 5 or more hours*
Original	8. In the last week you were in school, on how many days did you go to physical education (PE) classes?<br>☐ 0 days ☐ 1 day ☐ 2 days ☐ 3 days ☐ 4 days ☐ 5 days
Forward	*Săptămâna trecută* la școală, în câte zile ai fost la ore de educație fizică și sport?<br>*0/1 zi /2 zile/3 zile/4 zile/5 zile*
Backward	*Last week* at school, how many days did you go to physical education classes?<br>*0 / 1 Day / 2 days / 3 days / 4 days / 5 days*

Original	9. In the last week you were in school, the **MAIN** part of your journey to school was by:<br>☐ walking<br>☐ bicycle, roller-blade, skateboard or scooter<br>☐ bus, train, tram, underground or boat<br>☐ car, motorcycle or moped<br>☐ other ______________
Adapted	Cum parcurgi CEA MAI MARE PARTE din drumul tău de acasă la școală?<br>*Merg pe jos / Cu bicicleta, trotineta (nu electrică), role sau skateboard / Cu autobuzul, trolebuzul, tramvaiul sau alt mijloc de transport în comun / Cu mașina, motocicleta, motoreta, trotineta electrică / Alte variante*
Backward	How do you travel most of your way from home to school?<br>*Walk/by bicycle, scooter (not electric), roller skates or skateboard/by bus, trolleybus, tram or other means of public transport/by car, motorcycle, moped, electric scooter/other options*
Original	*(none)*
Adapted	Dacă ai răspuns „Alte variante” la întrebarea anterioară, te rugăm să explici:
Backward	If you answered "other" to the previous question, please explain:
Original	10. In the last week you were in school, **HOW LONG** did it take you to travel to school?<br>☐ < 5 minutes ☐ 5 - 15 minutes ☐ 16 - 30 minutes ☐ 31 minutes to 1 hour ☐ >1 hour
Forward	Cam cât timp durează drumul tău de acasă până la școală?<br>*Mai puțin de 5 minute / 5-15 minute / 16-30 de minute / Între 30 de minute și o oră / Peste 1 oră*
Backward	How long does it take to get from home to school?<br>*Less than 5 minutes / 5-15 minutes / 16-30 minutes / between 30 minutes and an hour / over 1 hour*
Original	11. During the past year (12 months), did you do any of these activities? (Check all that apply)<br>☐ sports teams ☐ dance / martial arts class ☐ art / music class ☐ none of these
Forward	La care dintre următoarele activități ai participat în ultimele 12 luni?<br>*Jocuri sportive de echipă / Dans, arte marțiale / Artă, muzică / Nici una dintre cele de mai sus*
Backward	Which of the following activities have you participated in in the last 12 months?<br>*Team sports / dance, martial arts / art, music / none of the above*
Original	12. During the past week (7 days), on how many days were you physically active for a total of at least 60 minutes per day? (all the time you spent in activities that increased your heart rate and made you breathe hard)<br>☐ 0 days ☐ 1 day ☐ 2 days ☐ 3 days ☐ 4 days ☐ 5 days ☐ 6 days ☐ 7 days
Forward	În ultimele 7 zile, în câte zile ai fost activ fizic pentru cel puțin 60 de minute pe zi? (activități care îți fac inima să bată mai repede și te fac să respiri mai repede ca de obicei)<br>*0 / 1 zi / 2 zile / 3 zile / 4 zile / 5 zile / 6 zile / 7 zile*
Backward	In the last 7 days, how many days have you been physically active for at least 60 minutes a day? (activities that make your heart beat faster and make you breathe faster than usual)<br>*0 / 1 Day / 2 days / 3 days / 4 days / 5 days / 6 days / 7 days*
Original	**Please tick the box that most sounds like you:**<br>**Disagree a Lot → Agree a Lot**<br>**1 2 3 4 5**
Forward	Pentru fiecare dintre următoarele 8 afirmații, te rugăm selectează un răspuns între 1 și 5:<br>1 = dacă ești în TOTAL DEZACORD cu afirmația respectivă<br>2 = dacă ești în DEZACORD cu afirmația respectivă<br>3 = dacă ești INDECIS/Ă privind afirmația respectivă<br>4 = dacă ești de ACORD cu afirmația respectivă<br>5 = dacă ești TOTAL DE ACORD cu afirmația respectivă
Backward	For each of the following 8 statements, please select a response between 1 and 5:<br>1 = if you completely disagree with the statement<br>2 = if you disagree with the statement<br>3 = if you are undecided about the statement<br>4 = if you agree with the statement<br>5 = if you fully agree with the statement

Original	13. I can be physically active during my free time on most days.
Forward	Aleg să fiu activ fizic în timpul meu liber în majoritatea zilelor.
Backward	I choose to be physically active in my free time most days.
Original	14. I can ask my parent or other adult to do physically active things with me.
Forward	Pot să îmi rog părinții sau alți adulți să facă activități fizice cu mine.
Backward	I can ask my parents or other adults to do physical activities with me.
Original	15. I can be physically active during my free time on most days even if I could watch TV or play video games instead.
Adapted	Prefer să fiu activ fizic în timpul meu liber în majoritatea zilelor, chiar dacă în loc de activități fizice aș putea să mă uit la televizor sau să mă joc jocuri video.
Backward	I prefer to be physically active in my free time most days, even though instead of physical activities I could watch TV or play video games.
Original	16. I can be physically active during my free time on most days even if it is very hot or cold outside.
Forward	Prefer să fiu activ fizic în timpul meu liber în majoritatea zilelor, chiar dacă este foarte cald sau foarte frig afară.
Backward	I prefer to be physically active in my free time most days, even if it is very hot or very cold outside.
Original	17. I can ask my best friend to be physically active with me during my free time on most days.
Forward	Pot să îl rog pe prietenul meu cel mai bun să facă activități fizice cu mine în timpul meu liber în majoritatea zilelor.
Backward	I can ask my best friend to do physical activities with me in my spare time most days.
Original	18. I can be physically active during my free time on most days even if I have to stay at home.
Forward	Prefer să fiu activ fizic în timpul meu liber în majoritatea zilelor, chiar dacă trebuie să stau acasă
Backward	I prefer to be physically active in my free time most days, even if I have to stay at home
Original	19. I have the coordination I need to be physically active during my free time on most days.
Forward	Am coordonarea și motivația necesară pentru a fi activ fizic în timpul meu liber în majoritatea zilelor.
Backward	I have the coordination and motivation to be physically active in my free time most days.
Original	20. I can be physically active during my free time on most days no matter how busy my day is.
Forward	Prefer să fiu activ fizic în timpul meu liber în majoritatea zilelor indiferent cât de ocupată mi-a fost ziua.
Backward	I prefer to be physically active in my free time most days no matter how busy my day has been.
Original	**There are lots of reasons why people take part in physical activity. Please tick the box to show how much each of the reasons below is true for you:**<br>never true for me / a little bit true for me / sometimes true for me / true for me / very true for me
Forward	Există multe motive pentru care oamenii participă la activități fizice. Pentru fiecare dintre următoarele 5 afirmații, te rugăm selectează un răspuns între 1 și 5:<br>1 = dacă afirmația NU ESTE NICIODATĂ ADEVĂRATĂ pentru tine<br>2 = dacă afirmația ESTE RAREORI ADEVĂRATĂ pentru tine<br>3 = dacă afirmația ESTE UNEORI ADEVĂRATĂ pentru tine<br>4 = dacă afirmația ESTE DESEORI ADEVĂRATĂ pentru tine<br>5 = dacă afirmația este ÎNTOTDEAUNA ADEVĂRATĂ pentru tine
Backward	There are many reasons why people participate in physical activities. For each of the following 5 statements, please select a response between 1 and 5:<br>1 = if the statement is never true to you<br>2 = if the statement is rarely true to you<br>3 = if the statement is sometimes true for you

	4 = if the statement is often true for you<br>5 = if the statement is always true for you
Original	21. I take part in exercise because other people say I should
Forward	Iau parte la exerciții fizice pentru că alți oameni îmi spun că ar trebui să fac asta.
Backward	I take part in exercise because other people tell me I should do it.
Original	22. It's important to me to exercise regularly
Forward	Este important pentru mine să fac exerciții fizice în mod regulat.
Backward	It is important for me to exercise regularly.
Original	23. I can't see why I should bother exercising
Forward	Nu văd de ce ar trebui să mă deranjez să fac exerciții fizice.
Backward	I don't see why I should bother exercising.
Original	24. I feel like a failure when I haven't exercised in a while
Forward	Am senzația de eșec atunci când nu am făcut exerciții fizice o perioadă de timp.
Backward	I have the feeling of failure when I have not exercised for a period of time.
Original	25. I find exercise a pleasurable activity
Forward	Pentru mine exercițiile fizice sunt o activitate plăcută
Backward	For me exercise is a pleasant activity
Original	☐☐:☐☐ AM / PM (circle AM or PM)
Forward	Pentru următoarele 4 întrebări, notează ora și minutul care corespund cel mai bine cu situația ta. Te rugăm să folosești formatul de 24 de ore (vezi exemplul). De exemplu: dacă mă culc la 10 seara, atunci scriu ora 22:00
Backward	For the next 4 questions, write down the time and minute that best correspond to your situation. Please use the 24-hour format (see example). For example: if I go to bed at 10 o'clock in the evening, then I write 22:00
Original	**26.** During the past week, what time have you usually turned out the light and gone to sleep on school days?
Forward	*Săptămâna trecută*, la ce oră ai stins lumina și te-ai culcat în mod obișnuit în zilele de școală?
Backward	*Last week,* at what time did you typically turn off the lights and go to bed on school days?
Original	**27.** During the past week, at what time have you usually woken up in the morning on school days**?**
Forward	*Săptămâna trecută*, la ce oră te-ai trezit în mod obișnuit în zilele de școală?
Backward	*Last week,* at what time did you typically wake up on school days?
Original	**28.** During the past week, what time have you usually turned out the light and gone to sleep on weekend days?
Forward	*Săptămâna trecută*, la ce oră ai stins lumina și te-ai culcat în mod obișnuit în zilele de weekend?
Backward	*Last week,* at what time did you turn off the lights and go to bed on weekends?
Original	29. During the past week, at what time have you usually woken up in the morning on weekend days?
Forward	*Săptămâna trecută*, la ce oră te-ai trezit în mod obișnuit în zilele de weekend?
Backward	*Last week,* at what time did you typically wake up on weekends?
Original	30. During the past week, how would you rate your sleep **quality** overall (how **well** you sleep)?<br>☐ very good ☐ fairly good ☐ fairly bad ☐ very bad
Forward	Cum apreciezi cât de bine ai dormit *săptămâna trecută*?<br>*Foarte bine / Destul de bine / Mediu / Destul de rău / Foarte rău*
Backward	How would you assess how well you slept last week?<br>*Very good / pretty good / medium / pretty bad / very bad*
Original	31. During the past week, how would you rate your sleep **quantity** overall (how **much** you sleep)?<br>☐ very good ☐ fairly good ☐ fairly bad ☐ very bad
Forward	Cum apreciezi cât ai dormit *săptămâna trecută*? (cât timp ai dormit)<br>*Foarte mult / Mult / Destul / Puțin / Foarte puțin*
Backward	How much would you say you slept last week? (how long have you slept)<br>*very much / much / enough / little / very little*

Original	32. Do you have a television in your bedroom?<br>☐ Yes ☐ No
Forward	Ai televizor în camera în care dormi?<br>*Da/Nu*
Backward	Do you have a TV in the room you sleep in?<br>*Yes/No*
Original	(none)
Forward	Ai calculator în camera în care dormi?<br>*Da/Nu*
Backward	Do you have a computer in the room you sleep in?<br>*Yes/No*

Original

33. How many times do you usually eat . . . ? (Please mark only one box for each line)

	Never	Less than once a week	Once a week	2-4 days a week	5-6 days a week	Once a day, every day	Every day, more than once
Fruits	☐	☐	☐	☐	☐	☐	☐
Vegetables	☐	☐	☐	☐	☐	☐	☐
Sweets (candy/chocolate)	☐	☐	☐	☐	☐	☐	☐
Regular cola or soft drinks that contain sugar	☐	☐	☐	☐	☐	☐	☐
Cake, pastries, or donuts	☐	☐	☐	☐	☐	☐	☐
Diet cola or diet soft drinks	☐	☐	☐	☐	☐	☐	☐
Potato chips	☐	☐	☐	☐	☐	☐	☐
French fries	☐	☐	☐	☐	☐	☐	☐
Dark green vegetables (broccoli, spinach, etc.)	☐	☐	☐	☐	☐	☐	☐
Orange vegetables (carrots, squash, sweet potato, etc.)	☐	☐	☐	☐	☐	☐	☐
Fruit juice	☐	☐	☐	☐	☐	☐	☐
Low fat milk (1%,2%, skim)	☐	☐	☐	☐	☐	☐	☐
Whole milk (homogenized)	☐	☐	☐	☐	☐	☐	☐
Cheese	☐	☐	☐	☐	☐	☐	☐
Other milk products (yogurt, chocolate milk, pudding, etc.)	☐	☐	☐	☐	☐	☐	☐
Whole grain bread or cereal (oatmeal, muesli, etc.)	☐	☐	☐	☐	☐	☐	☐
Meat alternatives (beans, lentils, tofu, eggs, peanut butter, etc.)	☐	☐	☐	☐	☐	☐	☐
Energy drinks (Red Bull, Rock Star, Guru, etc.)	☐	☐	☐	☐	☐	☐	☐
Sports drinks (Gatorade, Powerade, etc.)	☐	☐	☐	☐	☐	☐	☐
Fish	☐	☐	☐	☐	☐	☐	☐
Ice cream	☐	☐	☐	☐	☐	☐	☐
Fried food such as chicken wings, chicken fingers, etc.	☐	☐	☐	☐	☐	☐	☐
Fast foods such as pizza, hamburgers, etc.	☐	☐	☐	☐	☐	☐	☐

Forward	Câte zile pe săptămână consumi deobicei<br>Fructe / Legume / Dulciuri, bomboane, ciocolată / Sucuri care conțin zahăr / Prăjituri, patiserii, gogoși / Sucuri dietetice (fără zahăr) / Chipsuri din cartofi / Cartofi prăjiți / Legume verzi (broccoli, spanac, etc.) / Legume portocalii (morcov, dovleac, cartof dulce, etc.) / Suc de fructe

<table>
<tr><td></td><td>/ Lapte degresat (1-2% grăsime) / Lapte integral / Brânză / Alte lactate (iaurt, budincă, etc.) / Pâine integrală sau cereale integrale (ovăz, muesli, etc.) / Alternative la carne (fasole, linte, tofu, ouă, unt de arahide etc.) / Băuturi energizante (Red Bull, Monster, Hell, etc.) / Băuturi pentru sport (de ex. Gatorade) / Pește / Înghețată / Mâncare prăjită / Fast food (pizza, hamburgeri, etc.)
Niciodată / Mai puțin de o dată / O dată / 2-4 zile / 5-6 zile / O data pe zi, în fiecare zi / De mai multe ori pe zi, în fiecare zi</td></tr>
<tr><td>Backward</td><td>How many days a week do you usually eat
Fruits / vegetables / sweets, candy, chocolates / sugary juices / cakes, pastries, Donuts / sugar-free juices / potato chips / French fries / green vegetables (broccoli, spinach, etc.) / orange vegetables (carrot, pumpkin, sweet potato, etc.) / fruit juice / skim milk (1-2% fat) / whole milk / cheese / other dairy (yogurt, pudding, etc.) / whole or whole grain bread (oats, muesli, etc.) / meat alternatives (beans, lentils, tofu peanut butter, etc.) / energy drinks (Red Bull, Monster, hell, etc.) / sports drinks (eg. Gatorade) / fish / ice cream / fried food / Fast food (pizza, hamburgers, etc.)
Never / less than once / once / 2-4 days / 5-6 days / once a day, every day / several times a day, every day</td></tr>
<tr><td>Original</td><td>34. How many times do you usually eat the following food items while watching television?
<table>
<tr><th></th><th>Never</th><th>Less than once a week</th><th>Once a week</th><th>2-4 days a week</th><th>5-6 days a week</th><th>Once a day, every day</th><th>Every day, more than once</th></tr>
<tr><td>Potato chips or peanuts</td><td>☐</td><td>☐</td><td>☐</td><td>☐</td><td>☐</td><td>☐</td><td>☐</td></tr>
<tr><td>Fried food such as chicken wings, chicken fingers, french fries, etc.</td><td>☐</td><td>☐</td><td>☐</td><td>☐</td><td>☐</td><td>☐</td><td>☐</td></tr>
<tr><td>Cookies, biscuits, chocolate or candy bars</td><td>☐</td><td>☐</td><td>☐</td><td>☐</td><td>☐</td><td>☐</td><td>☐</td></tr>
<tr><td>Ice cream</td><td>☐</td><td>☐</td><td>☐</td><td>☐</td><td>☐</td><td>☐</td><td>☐</td></tr>
<tr><td>Fast foods such as pizza, hamburgers, etc.</td><td>☐</td><td>☐</td><td>☐</td><td>☐</td><td>☐</td><td>☐</td><td>☐</td></tr>
<tr><td>Fruits or vegetables</td><td>☐</td><td>☐</td><td>☐</td><td>☐</td><td>☐</td><td>☐</td><td>☐</td></tr>
</table></td></tr>
<tr><td>Forward</td><td>În câte zile pe săptămână mănânci de obicei următoarele în timp ce te uiți la un ecran (televizor sau calculator sau telefon)?
Chipsuri din cartofi / Alune sărate / Semințe de floarea soarelui / Mâncăruri prăjite (cartofi prăjiți, aripioare de pui, etc.) / Prăjituri, biscuiți, ciocolată / Înghețată / Fast food (pizza, hamburgeri, etc.) / Fructe sau legume / Nuci sau alune neprăjite și nesărate
Niciodată / Mai puțin de o dată / O dată / 2-4 zile / 5-6 zile / O data pe zi, în fiecare zi / De mai multe ori pe zi, în fiecare zi</td></tr>
<tr><td>Backward</td><td>How many days a week do you usually eat the following while looking at a screen (TV or computer or phone)?
Potato chips / salted peanuts / sunflower seeds / fried foods (French fries, chicken wings, etc.) / cakes, biscuits, chocolate / ice cream / Fast food (pizza, hamburgers, etc.) / fruits or vegetables / nuts or peanuts not roasted and unsalted
Never / less than once / once / 2-4 days / 5-6 days / once a day, every day / several times a day, every day</td></tr>
<tr><td>Original</td><td>35. How often do you usually have breakfast (more than a glass of milk or fruit juice)? Mark one box for <u>weekdays</u> and one box for <u>weekend</u>.
<table>
<tr><th><u>Weekdays</u></th><th><u>Weekend</u></th></tr>
<tr><td>☐ I never have breakfast on weekdays</td><td>☐ I never have breakfast on the weekend</td></tr>
<tr><td>☐ One day</td><td>☐ I usually have breakfast on only one day of the weekend (Saturday OR Sunday)</td></tr>
<tr><td>☐ Two days</td><td></td></tr>
<tr><td>☐ Three days</td><td>☐ I usually have breakfast on both weekend days (Saturday AND Sunday)</td></tr>
<tr><td>☐ Four days</td><td></td></tr>
<tr><td>☐ Five days</td><td></td></tr>
</table></td></tr>
<tr><td>Adapted</td><td>Cât de des iei micul dejun în zilele săptămânii, exceptând weekendul? (mai mult decât un pahar de lapte sau un pahar de suc de fructe)
Niciodată/ O zi pe săptămână / 2 zile pe săptămână / 3 zile pe săptămână / 4 zile pe săptămână / 5 zile pe săptămână</td></tr>
</table>

	Cât de des iei micul dejun **în zilele de weekend**? (mai mult decât un pahar de lapte sau un pahar de suc de fructe)<br>*De obicei niciodată / De obicei sâmbăta SAU duminica / De obicei și sâmbăta ȘI duminica*
Backward	How often do you eat breakfast **on weekdays**, except weekends? (more than a glass of milk or a glass of fruit juice)<br>*Never/ one day a Week / 2 days a Week / 3 days a Week / 4 days a Week / 5 days a week*<br><br>How often do you eat breakfast on weekends? (more than a glass of milk or a glass of fruit juice)<br>*Usually never / usually Saturday or Sunday / usually also Saturday and Sunday*
Original	36. Does your school serve school lunches?<br>☐ Yes ☐ No
Forward	Școala ta oferă masă de prânz pentru elevi?<br>*Da / Nu*
Backward	Does your school offer lunch to students?<br>*Yes / No*
Original	37. In the <u>last week</u> you were in school, about **how many times a week** did you eat a school lunch?<br>☐ 0 days ☐ 1 day ☐ 2 days ☐ 3 days ☐ 4 days ☐ 5 days
Forward	Săptămâna trecută, în câte zile ai mâncat la prânz la școală?<br>*Niciodată / O zi / 2 zile / 3 zile / 4 zile / 5 zile*
Backward	Last week, how many days did you eat lunch at school?<br>*Never / one day / 2 days / 3 days / 4 days / 5 days*
Original	38. During the <u>past week</u>, how many meals (breakfast, lunch or dinner) did you get that were **prepared away from home** in places such as restaurants, fast food places, food stands, grocery stores or vending machines? (please do not include meals provided as part of school breakfast or school lunch)<br>☐☐ meals
Forward	Săptămâna trecută, la câte mese în total (mic dejun, prânz sau cină) ai consumat produse care nu au fost pregătite în casă (de exemplu, la restaurant, fast food etc.)?
Backward	Last week, how many meals in total (breakfast, lunch or dinner) did you eat that were not prepared in the home (for example, at the restaurant, fast food, etc.)?
Original	**How well do these statements describe you?** (Put a mark in the box that best describes how often this happens).<br>Never or Almost Never / Sometimes / Usually or Always
Forward	Cât de bine ți se potrivesc următoarele afirmații?<br>*Niciodată sau aproape niciodată/ Câteodată / Deobicei sau întotdeauna*
Backward	How well do the following statements describe you?<br>*Never or almost never / sometimes / usually or always*
Original	39. When I am worried I eat more
Forward	Mănânc mai mult când sunt îngrijorat
Backward	I eat more when I'm worried
Original	40. I eat when I am mad
Forward	Mănânc când sunt furios
Backward	I eat when I'm angry
Original	41. When I do something well I give myself a food treat
Forward	Când fac ceva bine, mă recompensez cu mâncare
Backward	When I do something right, I reward myself with food
Original	42. When I am sad I eat more
Forward	Mănânc mai mult când sunt trist
Backward	I eat more when I'm sad
Original	43. When I am happy I eat more
Forward	Mănânc mai mult când sunt fericit
Backward	I eat more when I'm happy

Original	44. When I am bored I eat more
Forward	Mănânc mai mult când sunt plictisit
Backward	I eat more when I'm bored
Original	**45.** I eat between meals even when I am not hungry
Forward	Mânânc între mese chiar dacă nu îmi este foame
Backward	I eat between meals even though I'm not hungry
Original	**Thinking about the last week…..** (Put a mark in the box that best describes how you felt)<br>Not at all Slightly Moderately Very Extremely
Forward	Dacă te gândești la săptămâna trecută...<br>*Deloc / Puțin / Moderat / Foarte / Extrem*
Backward	If you think about last week...<br>*Not At All / A little / Moderately / Very / Extremely*
Original	46. Have you felt fit and well?
Forward	Te-ai simțit bine și în formă?
Backward	Did you feel well and fit?
Original	47. Have you felt full of energy?
Forward	Te-ai simțit plin de energie?
Backward	Did you feel full of energy?
Original	48. Have you felt sad?
Forward	Te-ai simțit trist?
Backward	Did you feel sad?
Original	49. Have you felt lonely?
Forward	Te-ai simțit singur?
Backward	Did you feel lonely?
Original	50. Have you had enough time for yourself?
Forward	Ai avut destul timp pentru tine?
Backward	Have you had enough time for yourself?
Original	51. Have you been able to do the things that you want to do in your free time?
Forward	Ai făcut lucrurile pe care ai dorit să le faci în timpul tău liber?
Backward	Did you do the things you wanted to do in your free time?
Original	52. Have your parent(s) treated you fairly?
Forward	Te-au tratat bine părinții tăi?
Backward	Did your parents treat you well?
Original	53. Have you had fun with your friends?
Forward	Te-ai distrat cu prietenii tăi?
Backward	Did you have fun with your friends?
Original	54. Have you got on well at school?
Forward	Te-ai descurcat la școală?
Backward	Did you do well at school?
Original	55. Have you been able to pay attention?
Adapted	Ai reușit să fii atent la școală?
Backward	Did you manage to pay attention at school?
Original	56. In general, how would you say your health is?<br>☐ excellent ☐ very good ☐ good ☐ fair ☐ poor
Adapted	În general, cum apreciezi că este sănătatea ta?<br>*Excelentă / Foarte bună / Bună / Acceptabilă / Rea / Foarte rea*
Backward	In general, how would you rate your health?<br>*Excellent / very good / good / acceptable / bad / very bad*
	**Question, title or description**

Original	**ISCOLE Demographic and Family Health Questionnaire**
Adapted	Chestionar pentru părinți/tutori - studiul obezității
Backward	Questionnaire for parents / guardians - Obesity study
Original	(none)
Adapted	Vă rugăm să citiți fiecare întrebare cu atenție.<br>Marcați la fiecare întrebare răspunsul care se potrivește cel mai bine.<br>De ținut minte:<br>- Acesta nu este un test, așa că nu există răspunsuri greșite.<br>- Datele obținute au caracter confidențial
Backward	Please read each question carefully.<br>Mark each question with the answer that suits you best.<br>Remember:<br>- This is not a test, so there are no wrong answers.<br>- The data obtained are confidential
Original	**A. GENERAL INFORMATION**
Adapted	(none)
Original	Child's Name: ____ Last First Middle
Adapted	Care sunt numele și prenumele copilului aflat în grija dumneavoastră care a fost inclus în acest studiu?
Backward	What is the name and surname of the child in your care who was included in this study?
Original	(none)
Adapted	Dacă aveți mai mulți copii incluși în studiu, vă rugăm să completați câte un chestionar pentru fiecare dintre aceștia.
Backward	If you have several children included in the study, please fill out a questionnaire for each of them.
Original	Parent's or Guardian's Name: ____ Last First Middle
Adapted	Vă rugăm completați numele și prenumele dumneavoastră
Backward	Please fill in your first and last name
Original	Home Address: ____ Street Address Apt. # Town or City State Postal/Zip Code
Adapted	(removed)
Original	Nearest Cross-Street to Home: ____
Adapted	(removed)
Original	(none)
Adapted	În ce mediu se află locuința copilului?<br>*Urban/Rural*
Backward	What environment is the child's home in?<br>*Urban / Rural*
Original	Phone Number: ( ) ____ E-Mail: Area Code
Adapted	Vă rugăm să completați un număr de contact. *Numărul dumneavoastră de telefon va fi utilizat exclusiv pentru a fi contactați în legătură cu prelevarea datelor necesare acestui studiu.*
Backward	Please fill in a contact number. *Your telephone number will only be used to contact you in connection with the collection of data required for this study.*
Original	(none)
Adapted	Care este relația dumneavoastră cu copilul inclus în studiu?<br>*Mamă/Mamă vitregă/Bunică/Tată/Tată vitreg/ Bunic/Altă variantă*

Backward	What is your relationship with the child included in the study? Mother / stepmother / grandmother / father / stepfather / grandfather / other
Original	How long have you lived at the current address? ________years and _________months
Forward	(removed)
Original	**B. DEMOGRAPHICS OF CHILD**
Adapted	(removed)
Original	Birth date _____/_____/_____ dd/mmm/yyyy Example: 02/Jun/2011 Age _____years
Forward	Care este data nașterii copilului?
Backward	What is the child's date of birth?
Original	Gender: ☐ Male ☐ Female
Forward	Care este genul copilului?<br>*Masculin/Feminin*
Backward	What is the child's gender?<br>*Male / Female*
Original	Ethnicity:<br>☐ White<br>☐ African American<br>☐ Asian<br>☐ American Indian, Aleutian, Alaska native or Eskimo<br>☐ Pacific Islander<br>☐ Don't know<br>☐ Other ____________________<br>Are you of Hispanic origin? ☐ Yes ☐ No
Adapted	(removed)
Original	In what country was the child born? _________________
Forward	În ce țară s-a născut copilul?
Backward	In which country was the child born?
Original	How many biological brothers and sisters does the child have?_______________
Adapted	Câți frați și câte surori are copilul aflat în grija dumneavoastră? (bifați două căsuțe)<br>*Nicio soră/O soră/Două surori/Trei surori/Patru surori/Cinci surori/Mai mult de cinci surori*<br>*Niciun frate/Un frate/Doi frați/Trei frați/Patru frați/Cinci frați/Mai mult de cinci frați*
Backward	How many brothers and sisters has the child in your care got? (check two boxes)<br>*no sister/one sister/two sisters/three sisters/four sisters/five sisters/more than five sisters No brother / one brother / two brothers / Three Brothers / Four Brothers / five brothers/more than five brothers*
Original	What are their ages? _____yrs _____yrs _____yrs _____yrs _____yrs<br>_____yrs _____yrs _____yrs _____yrs _____yrs
Adapted	Dacă copilul aflat în grija dumneavoastră are frați sau surori, vă rugăm să scrieți pentru fiecare dintre aceștia vârsta, greutatea și înălțimea în următorul format:<br>Frate 1 - Vârstă (ani) - Greutate (kg) - Înălțime (cm)
Backward	If the child in your care has got brothers or sisters, please write for each of them the age, weight and height in the following format: Brother 1-Age (Years) - Weight (kg) - height (cm)
Original	(none)
Adapted	La ce școală a fost înscris copilul în clasele V-VIII?
Backward	What school was your child enrolled in during grades V-VIII?

Original	(none)
Adapted	În ce clasă este copilul?
Backward	What grade is the child in?
Original	**C. HEALTH HISTORY OF CHILD**
Adapted	(removed)
Original	**1.** Birth Weight: _______kg OR _______lbs &_____oz Birth Length: ____cm OR ____inches
Forward	Care a fost greutatea în grame la naștere a copilului?
Backward	What was the child's birthweight in grams?
Original	**2.** Length of Pregnancy: _______weeks OR _____months
Forward	Copilul a fost născut la termen?<br>*Da/Nu – Prematur/Nu - Sarcină suprapurtată*<br>Dacă la întrebarea precedentă ați răspuns NU, vă rugăm precizați durata sarcinii în săptămâni
Backward	Was the baby born at term?<br>*Yes / no-premature / no -– pregnancypost-term*<br>If you answered No to the previous question, please specify the duration of pregnancy in weeks.
Original	**3.** Did mother develop gestational diabetes during pregnancy with **THIS** child? ☐ Yes No ☐
Forward	A dezvoltat mama copilului pe parcursul sarcinii diabet gestațional?<br>***Da/Nu***
Backward	Did the mother of the child develop gestational diabetes during pregnancy?<br>*Yes/No*
Original	**4.** Fed breast milk? ☐ Yes ☐ No If No, please skip to question 5.
Forward	Copilul a fost alăptat?<br>*Da/Nu*
Backward	Was the baby breastfed?<br>*Yes/No*
Original	Age when **COMPLETELY** stopped being fed breast milk: _____months
Forward	Dacă la întrebarea precedentă ați răspuns DA, la ce vârstă (în luni) s-a oprit din alăptat copilul?
Backward	If you answered yes to the previous question, at what age (in months) did the baby stop breastfeeding?
Original	Age when **FIRST** fed formula: _____months
Adapted	Copilul a fost hrănit cu formulă?<br>*Da/Nu*<br>Dacă la întrebarea precedentă răspunsul a fost DA, la ce vârstă în luni ați început să hrăniți copilul cu formulă?
Backward	Was the baby formula fed?<br>*Yes/No*<br>If the answer was yes for the previous question, at what age in months did you start feeding the baby formula?
Original	**5.** Age when **COMPLETELY** stopped drinking formula: _____months
Forward	Dacă ați hrănit copilul cu formulă, la ce vârstă în luni ați încetat hrănirea cu formulă?
Backward	If you fed your baby formula, at what age in months did you stop formula feeding?
Original	**C. FAMILY DEMOGRAPHICS AND HEALTH**
Adapted	(removed)

Original	**6.** What is the marital status of the child's parents?<br>☐ Married<br>☐ Divorced or separated<br>☐ Never married<br>☐ Widowed parent
Forward	Care este starea civilă a părinților copilului?<br>*Căsătoriți/Divorțați sau separate/Niciodată căsătoriți/Văduv/ă*
Backward	What is the marital status of the child's parents?<br>*Married / divorced or separated/never married/widowed*
Original	**7.** How many people live in your household (at this address)? ____<br>**7a.** Who lives with the child **at this address** (check all that apply)?<br>☐ Biological Mother ☐ Brother(s) or Sister(s)<br>☐ Biological Father ☐ Grandparent(s)<br>☐ Adoptive Mother ☐ Other Relative(s)<br>☐ Adoptive Father ☐ Friend(s)<br>☐ Step Mother ☐ Legal Guardian(s)<br>☐ Step Father ☐ OTHER
Forward	Câte persoane locuiesc împreună cu copilul aflat în grija dumneavoastră?<br>*Mama biologică/Mama vitregă/Tatăl biologic/Tatăl vitreg/Frați și surori/Bunici/Prieteni/Alte rude/Alte persoane*
Backward	How many people live with the child in your care?<br>*Biological mother/stepmother/biological father/stepfather/brothers and sisters/grandparents/friends/ other relatives/other people*
Original	**8.** What is the **COMBINED** annual income for your household (before taxes)?<br>☐ Less than $10,000<br>☐ $10,000 - $29,999<br>☐ $30,000 - $49,999<br>☐ $50,000 - $69,999<br>☐ $70,000 - $89,999<br>☐ $90,000 - $109,999<br>☐ $110,000 - $139,999<br>☐ $140,000 and above
Forward	În ce categorie apreciați că se încadrează în prezent venitul dumneavoastră familial lunar?<br>*Sub medie/Mediu/Peste medie*
Backward	What category do you think your monthly family income currently falls into?<br>*Below average/average/above average*
Original	**9.** How many functioning motorized vehicles (car, truck, motorcycle, moped, etc.) are available for use at your house?<br>☐ 0<br>☐ 1<br>☐ 2<br>☐ 3<br>☐ 4<br>☐ 5 or more
Forward	Câte vehicule motorizate (autoturisme, motociclete, scutere etc.) aveți în posesie sau la dispoziție?
Backward	How many motorized vehicles (cars, motorcycles, scooters, etc.) do you have in your possession or at your disposal?

Original	**10.** How many television sets are in your household?<br>☐ 0<br>☐ 1<br>☐ 2<br>☐ 3<br>☐ 4<br>☐ 5 or more
Forward	Câte televizoare sunt disponibile la domiciliul copilului aflat în grija dumneavoastră?
Backward	How many TVs are available at the home of the child in your care?
Original	**11.** What best describes your type of television service for the **primary** television in the house?<br>☐ No television<br>☐ Antenna only<br>☐ Basic cable<br>☐ Cable + premium channel(s)<br>☐ Satellite dish<br>☐ Other<br>☐ Don't know
Adapted	(removed)
Original	**12.** What best describes your type of internet service?<br>☐ No internet access<br>☐ Dial-up modem<br>☐ DSL modem<br>☐ Cable modem<br>☐ Other<br>☐ Don't know
Adapted	(removed)
Original	**13.** What is the **MOTHER'S** highest level of education completed?<br>☐ Less than high school<br>☐ Some high school<br>☐ High school diploma/GED<br>☐ Associate's degree or 1-3 years of college<br>☐ Bachelor's degree<br>☐ Graduate/professional degree
Forward	Care este nivelul de educație cel mai înalt absolvit cu succes al mamei copilului?<br>*Fără școală absolvită / Învățământ primar (clase 1-4) / Învățământ gimnazial (clase 5-8)/ Liceu/școală profesională / Școală postliceală / Învățământ universitar / Master / Doctorat / Studii postdoctorale*
Backward	What is the highest completed level of education of the child's mother? No Graduate School / Primary Education (grades 1-4) / secondary education (grades 5-8)<br>*High school / vocational school / post-secondary school / university education / Master's / doctoral / postdoctoral studies*
Original	**14.** How many hours per week does the **MOTHER** work outside the home?<br>☐ None<br>☐ Less than 15 hours/week<br>☐ 15-35 hours per week<br>☐ Full time (36+ hours per week)
Forward	Câte ore lucrează mama copilului în afara domiciliului?<br>*0 (casnică) / Mai puțin de 20 de ore/săptămână / 20 de ore pe săptămână (jumătate de normă) / 40 de ore pe săptămână (normă întreagă) / Peste 40 de ore pe săptămână*
Backward	How many hours does the child's mother work outside the home?<br>*0 (housewife) / less than 20 hours/week / 20 hours per week (part time) / 40 hours per week (full time) / over 40 hours per week*

Original	**15.** What is the **FATHER'S** highest level of education completed?<br>☐ Less than high school<br>☐ Some high school<br>☐ High school diploma/GED<br>☐ Associate's degree or 1-3 years of college<br>☐ Bachelor's degree<br>☐ Graduate/professional degree
Forward	Care este nivelul de educație cel mai înalt absolvit cu succes al tatălui copilului?<br>*Fără școală absolvită / Învățământ primar (clase 1-4) / Învățământ gimnazial (clase 5-8)/ Liceu/școală profesională / Școală postliceală / Învățământ universitar / Master / Doctorat / Studii postdoctorale*
Backward	What is the highest completed level of education of the child's father?<br>*High school / vocational school / post-secondary school / university education / Master's / doctoral / postdoctoral studies*
Original	**16.** How many hours per week does the **FATHER** work outside the home?<br>☐ None<br>☐ Less than 15 hours/week<br>☐ 15-35 hours per week<br>☐ Full time (36+ hours per week)
Forward	Câte ore lucrează tatăl copilului în afara domiciliului?<br>*0 (casnic) / Mai puțin de 20 de ore/săptămână / 20 de ore pe săptămână (jumătate de normă) / 40 de ore pe săptămână (normă întreagă) / Peste 40 de ore pe săptămână*
Backward	How many hours does the child's father work outside the home?<br>*0 (domestic) / less than 20 hours / week / 20 hours per week (part time) / 40 hours per week (full time) / over 40 hours per week*
Original	**17.** Is this child adopted? ☐ Yes ☐ No
Adapted	(removed)
Original	**18.** Please answer the following questions with regard to the child's **BIOLOGICAL MOTHER**:<br>Current height: _____cm **or** _____feet and _______inches Current weight: _______kg **or** _______lbs<br>Current Age: _____ years<br>Age at child's birth: ____ years<br>☐ Biological Mother's information cannot be estimated or is not known
Forward	Care este vârsta actuală a mamei copilului?<br>Care este greutatea actuală (în kilograme) a mamei copilului?<br>Care este înălțimea actuală (în centimetri) a mamei copilului?
Backward	What is the current age of the child's mother?<br>What is the current weight (in kilograms) of the child's mother?<br>What is the current height (in centimeters) of the child's mother?
Original	**19.** Please answer the following questions with regard to the child's **BIOLOGICAL FATHER:**<br>Current height: _____cm **or** _____feet and _______inches Current weight: _______kg **or** _______lbs<br>Current age: _____ years<br>☐ Biological Father's information cannot be estimated or is not known
Forward	Care este vârsta actuală a tatălui copilului?<br>Care este greutatea actuală (în kilograme) a tatălui copilului?<br>Care este înălțimea actuală (în centimetri) a tatălui copilului?
Backward	What is the current age of the child's father?<br>What is the current weight (in kilograms) of the child's father?<br>What is the current height (in centimeters) of the child's father?

	**Question, title or description**
Original	Child feeding questionnaire
Adapted	Chestionar pentru părinți/tutori - studiul obezității

Backward	Questionnaire for parents / guardians - Obesity study
Original	1 When your child is at home, how often are you responsible for feeding her?
Forward	Când copilul aflat în grija dumneavoastră este acasă, cât de frecvent sunteți responsabil/ă pentru hrănirea acestuia?
Backward	When your child is at home, how often are you responsible for feeding him/her?
Original	2 How often are you responsible for deciding what your child's portion sizes are?
Forward	Cât de frecvent sunteți responsabil/ă să decideți **cât de mari sunt porțiile** pe care la mănâncă copilul aflat în grija dumneavoastră?
Backward	How often are you responsible for deciding the size of the portions your child eats?
Original	3 How often are you responsible for deciding if your child has eaten the right kind of foods?
Adapted	Cât de frecvent sunteți responsabil/ă să decideți dacă este potrivit tipul de mâncare pe care îl consumă copilul aflat în grija dumneavoastră?
Backward	How often are you responsible for deciding whether the type of food your child eats is appropriate?
Original answer options for questions 1-3	1 = never;<br>2 = seldom;<br>3 = half of the time;<br>4 = most of the time;<br>5 = always
Forward	1 – Niciodată; 2 – Rareori; 3 – În jumătate din ocazii; 4 – Deseori; 5 – Întotdeauna
Backward	1 – Never; 2 – Rarely; 3 – On half the occasions; 4 – Often; 5 – Always
Original	Perceived parent weight<br>4 Your Childhood (5 to 10 years old)<br>5 Your adolescence<br>6 Your 20s<br>7 At present<br>1 = markedly underweight;<br>2 = underweight;<br>3 = normal;<br>4 = overweight;<br>5 = markedly overweight
Adapted	Cum percepeți greutatea **dumneavoastră** în următoarele perioade:<br>Copilărie / Adolescență / Prezent<br>*Subpondere / Normopondere / Suprapondere / Obezitate / Obezitate extremă*
Backward	How do you perceive **your** weight during the following periods:<br>Childhood / Adolescence / Present<br>*Underweight / Normoweight / Overweight / Obesity / Extreme obesity*
Original	Perceived child weight<br>8 Your child during the first year of life<br>9 Your child as a toddler<br>10 Your child as a pre-schooler<br>11 Your child kindergarten through 2nd grade<br>12 Your from child 3rd through 5th grade<br>13 Your child from 6th through 8th grade<br>1 = markedly underweight;<br>2 = underweight;<br>3 = normal;<br>4 = overweight;<br>5 = markedly overweight
Adapted	Cum percepeți greutatea copilului în următoarele perioade:<br>Primul an de viață / 2-3 ani / Preșcolar / Clasele primare / Gimnaziu / Liceu<br>*Subpondere / Normopondere / Suprapondere / Obezitate / Obezitate extremă*
Backward	How do you perceive the weight of the **child** during the following periods:<br>First year of life / 2-3 years / preschool / primary classes / Middle School / High School<br>*Underweight / Normopondere / overweight / obesity / extreme obesity*
Original	14 How concerned are you about your child eating too much when you are not around her?<br>16 How concerned are you about your child becoming over weight?
Forward	Cât de îngrijorat/ă sunteți în legătură cu greutatea copilului aflat în grija dumneavoastră?
Backward	How worried are you about the weight of the child in your care?

Original	15 How concerned are you about your child having to diet to maintain a desirable weight?
Forward	Cât de îngrijorat/ă sunteți în legătură cu faptul că copilul aflat în grija dumneavoastră ar putea fi nevoit să urmeze o dietă pentru a atinge și menține o greutate normală?
Backward	How worried are you that your child may need to follow a diet to achieve and maintain a normal weight?
Original answer options for questions 14-16	1 = unconcerned;<br>2 = a little concerned;<br>3 = concerned;<br>4 = fairly concerned;<br>5 = very concerned
Adapted	DELOC ÎNGRIJORAT/Ă (1) - FOARTE ÎNGRIJORAT/Ă (5)
Backward	NOT AT ALL WORRIED (1) - VERY WORRIED (5)
Original	17 I have to be sure that my child does not eat too many sweets (candy, icecream, cake or pastries)
Forward	Trebuie să mă asigur că copilul aflat în grija mea nu consumă prea multe dulciuri (ciocolată, înghețată, bomboane, patiserii) sau sucuri cu zahăr.
Backward	I have to make sure that the child in my care does not consume too many sweets (chocolate, ice cream, candy, pastries) or sugary juices.
Original	18 I have to be sure that my child does not eat too many high-fat foods
Forward	Trebuie să mă asigur că copilul aflat în grija mea nu consumă prea multe alimente bogate în grăsimi.
Backward	I have to make sure that the child in my care does not consume too many high-fat foods.
Original	19 I have to be sure that my child does not eat too much of her favorite foods
Forward	Trebuie să mă asigur că copilul aflat în grija mea nu consumă prea mult din alimentele lui/ei preferate, oricare ar fi acestea.
Backward	I must make sure that the child in my care does not consume too much of his/her favorite foods, whatever they may be.
Original	20 I intentionally keep some foods out of my child's reach
Forward	Țin în mod intenționat anumite alimente ascunse sau în afara accesului copilului aflat în grija mea.
Backward	I intentionally keep certain foods hidden or out of the access of the child in my care.
Original	21 I offer sweets (candy, ice cream, cake, pastries) to my child as a reward for good behavior
Adapted	Ofer recompense sub formă de dulciuri sau sucuri îndulcite cu zahăr copilului aflat în grija mea pentru bună-purtare.<br>*1– Niciodată 2 – Rareori 3 – Uneori 4 – Frecvent 5 – Întotdeauna*
Backward	I offer rewards in the form of sweets or sugar-sweetened juices to the child in my care for good behavior.<br>*1-Never 2-Rarely 3 - Sometimes 4-Frequently 5-Always*
Original	22 I offer my child her favorite foods in exchange for good behavior
Forward	Ofer recompense sub forma mâncarurilor preferate copilului aflat în grija mea pentru bună-purtare.<br>*1– Niciodată 2 – Rareori 3 – Uneori 4 – Frecvent 5 – Întotdeauna*
Backward	I offer rewards in the form of favorite foods to the child in my care for good behavior.

	1-Never 2-Rarely 3 - Sometimes 4-Frequently 5-Always
Original	23 If I did not guide or regulate my child's eating, she would eat too many junk foods
Forward	Dacă nu aș avea grijă ce mănâncă copilul aflat în grija mea, acesta ar consuma prea multe mâncăruri nesănătoase.
Backward	If I did not take care of what the child in my care eats, s/he would consume too many unhealthy foods.
Original	24 If I did not guide or regulate my child's eating, she would eat too much of her favorite foods
Adapted	(removed)
Original	25 My child should always eat all of the food on her plate
Forward	Copilul aflat în grija mea trebuie să mănânce tot din farfurie.
Backward	The child in my care must eat everything from the plate.
Original	26 I have to be especially careful to make sure my child eats enough
Adapted	Trebuie să fiu atent/ă ca copilul să mănânce suficient la masă.
Backward	I need to be careful that the child eats enough.
Original	27 If my child says "I'm not hungry", I try to get her to eat anyway
Forward	Încerc să conving copilul să mănânce când e ora mesei, chiar dacă acesta spune că nu îi este foame.
Backward	I try to convince the child to eat when it's mealtime, even if s/he says s/he's not hungry.
Original	28 If I did not guide or regulate my child's eating, she would eat much less than she should
Forward	Dacă nu aș ghida alimentația copilului, acesta ar mânca mult mai puțin decât îi este necesar.
Backward	If I did not guide the child's nutrition, s/he would eat much less than s/he needs.
Original	(none)
Adapted	Dacă nu aș ghida alimentația copilului, acesta ar mânca mult mai mult decât îi este necesar.
Backward	If I did not guide the child's nutrition, s/he would eat much more than s/he needs.
Original answer options for questions 17-28	1 = disagree;<br>2 = slightly disagree;<br>3 = neutral;<br>4 = slightly agree;<br>5 = agree
Forward (kept for questions 17, 18, 23- 28 and newly added question)	1 = dacă sunteți în TOTAL DEZACORD cu afirmația respectivă<br>2 = dacă sunteți în DEZACORD cu afirmația respectivă<br>3 = dacă sunteți INDECIS/Ă privind afirmația respectivă<br>4 = dacă sunteți DE ACORD cu afirmația respectivă<br>5 = dacă sunteți TOTAL DE ACORD cu afirmația respectivă
Backward	1 = if you TOTALLY DISAGREE with that statement<br>2 = if you DISAGREE with that statement<br>3 = if you are UNDECIDED about that statement<br>4 = if you AGREE with that statement<br>5 = if you TOTALLY AGREE with that statement
Original	29 How much do you keep track of the sweets (candy, ice cream cake, pies, pastries)

Forward	Cât de frecvent monitorizați consumul de dulciuri (ciocolată, înghețată, bomboane, patiserii) sau de sucuri îndulcite cu zahăr al copilului aflat în grija dumneavoastră?
Backward	How frequently do you monitor the consumption of sweets (chocolate, ice cream, candy, pastries) or sugar-sweetened juices of the child in your care?
Original	30 How much do you keep track of the snack food (potato chips, Doritos, cheese puffs) that your child eats?
Forward	Cât de frecvent monitorizați consumul de alimente de tip snacks-uri (chips-uri de cartofi, pufuleți, etc.) al copilului aflat în grija dumneavoastră?
Backward	How frequently do you monitor the consumption of snack foods (potato chips, puffs, etc.) of the child in your care?
Original	31 How much do you keep track of the high-fat foods that your child eats?
Forward	Cât de frecvent monitorizați consumul de alimente cu conținut înalt de grăsimi al copilului aflat în grija dumneavoastră?
Backward	How frequently do you monitor the consumption of high-fat foods by the child in your care?
Original answer options for questions 29-31	1 = never;<br>2 = rarely;<br>3 = sometimes;<br>4 = mostly;<br>5 = always
Adapted	1 – Niciodată; 2 – Rareori; 3 – În jumătate din ocazii; 4 – Deseori; 5 – Întotdeauna
Backward	1 – Never; 2 – rarely; 3 – On half the occasions; 4 – Often; 5 – Always
Original	**ISCOLE NEIGHBORHOOD & HOME ENVIRONMENT QUESTIONNAIRE**
Adapted	Chestionar pentru părinți/tutori - studiul obezității
Backward	Questionnaire for parents / guardians - Obesity study
Original	Where "child" is mentioned, please respond only about the child who is participating in this study. Be as accurate as you can. There are no right or wrong answers. All information is strictly confidential.
Adapted	(see initial instructions for merged questionnaire)

Original

A. NEIGHBORHOOD COHESION

Do you agree or disagree with the following statements?	Strongly disagree	Somewhat disagree	Neutral	Somewhat agree	Strongly agree
1. People around my neighborhood are willing to help their neighbors.	○	○	○	○	○
2. This is a close-knit neighborhood.	○	○	○	○	○
3. People in my neighborhood can be trusted.	○	○	○	○	○
4. People in my neighborhood generally don't get along with each other.	○	○	○	○	○
5. People in my neighborhood do not share the same values, attitudes or beliefs.	○	○	○	○	○

B. NEIGHBORS AND FRIENDS

1. Think about the neighborhood or area in which you live. In general, how well do you feel you know your neighbors?

Not at all	Just a little	Moderately well	Extremely well
○	○	○	○

2. About how often do you talk to or visit with your immediate neighbors (people in the 10-20 households that live closest to you)?

Never	Once a year or less	Several times a year	Once a month	Several times a month	Several times a week	Almost every day
○	○	○	○	○	○	○

C. NEIGHBORHOOD RESPONSE

For the following statements, please mark how likely a neighbor would respond to, or take action in the following situations:	Very unlikely	Unlikely	Neither likely nor unlikely	Likely	Very likely
1. If a group of neighborhood children were skipping school and hanging out on a street corner, how likely is it that your neighbors would do something about it?	○	○	○	○	○
2. If some children were spray-painting graffiti on a local building, how likely is it that your neighbors would do something about it?	○	○	○	○	○
3. If a child was showing disrespect to an adult, how likely is it that people in your neighborhood would scold that child?	○	○	○	○	○
4. If there was a fight in front of your house and someone was being beaten or threatened, how likely is it that your neighbors would break it up?	○	○	○	○	○
5. Suppose that because of budget cuts the fire station closest to your home was going to be closed down by the city. How likely is it that neighborhood residents would organize to try to do something to keep the fire station open?	○	○	○	○	○

Adapted

(removed)

Original

D. FOODS IN THE HOME

How often are the following foods/drinks available in your home?	Never	Rarely	Sometimes	Often	Always
1. Chocolate candy	○	○	○	○	○
2. Other candy	○	○	○	○	○
3. Raw fruit (e.g., apples, oranges)	○	○	○	○	○
4. Cakes, brownies, muffins or cookies	○	○	○	○	○
5. Regular chips or crackers	○	○	○	○	○
6. Baked chips, low-fat crackers, pretzels	○	○	○	○	○
7. Raw vegetables (e.g., carrots)	○	○	○	○	○
8. 100% fruit juice	○	○	○	○	○
9. Juice drinks (e.g., Sunny delight)	○	○	○	○	○
10. Regular sodas with sugar	○	○	○	○	○
11. Diet or sugar free sodas	○	○	○	○	○
12. Sports drinks (e.g., Gatorade)	○	○	○	○	○
13. Fruit roll-ups or other dried fruit	○	○	○	○	○
14. Regular or 2% milk	○	○	○	○	○
15. 1% or fat-free milk	○	○	○	○	○
16. Sweetened breakfast cereal	○	○	○	○	○
17. Unsweetened breakfast cereal	○	○	○	○	○

Adapted

Cât de frecvent se regăsesc următoarele tipuri de alimente în locuința de domiciliu a copilului înscris în studiu?

Ciocolată / Alte dulciuri / Fructe crude / Fructe uscate / Torturi, prăjituri, patiserii / Chips-uri de cartofi, covrigei, pufuleți / Nuci și alune neprăjite și nesărate / Nuci și alune prăjite și sărate / Legume crude / Sucuri de fructe 100% / Sucuri îndulcite cu zahăr / Sucuri fără zahăr / Lapte integral / Lapte degresat / Cereale cu zahăr / Cereale neîndulcite

<table>
<tr><td></td><td>Niciodată / Rareori / Uneori / Deseori / Întotdeauna</td></tr>
<tr><td>Backward</td><td>How frequently are the following types of food found in the home of the child enrolled in the study?
Chocolate / other sweets / raw fruits / dried fruits / cakes, cakes, pastries / potato chips, pretzels, puffs / unroasted and unsalted nuts and peanuts / roasted and salted nuts and peanuts / raw vegetables / 100% fruit juices / sweetened juices / sugar-free juices / whole milk / skimmed milk / sugary cereals / unsweetened cereals
Never / Rarely / Sometimes / Often / Always</td></tr>
<tr><td>Original</td><td>E. WHERE YOU SHOP
<table>
<tr><th>When you, or the main food shopper in your home, go food shopping, how often do you go to each of these types of stores?</th><th>Never</th><th>Rarely</th><th>Sometimes</th><th>Often</th><th>Always</th></tr>
<tr><td>1. Large supermarket or discount warehouse</td><td>○</td><td>○</td><td>○</td><td>○</td><td>○</td></tr>
<tr><td>2. Small to medium food store</td><td>○</td><td>○</td><td>○</td><td>○</td><td>○</td></tr>
<tr><td>3. Convenience store</td><td>○</td><td>○</td><td>○</td><td>○</td><td>○</td></tr>
<tr><td>4. Farmer's market/produce stand</td><td>○</td><td>○</td><td>○</td><td>○</td><td>○</td></tr>
<tr><td>5. Other, specify: ____________________</td><td>○</td><td>○</td><td>○</td><td>○</td><td>○</td></tr>
</table></td></tr>
<tr><td>Forward</td><td>Cât de frecvent faceți cumpărături de alimente de la următoarele tipuri de magazine?
Hipermarket-uri (Carrefour, Kaufland, Auchan, etc.) / Supermarket-uri de cartier / Aprozar Piață / Producători locali / Altele
Niciodată / Rareori / Uneori / Deseori / Întotdeauna</td></tr>
<tr><td>Backward</td><td>How frequently do you shop for groceries from the following types of stores? Hypermarkets (Carrefour, Kaufland, Auchan, etc.) / neighborhood supermarkets / grocery market / local producers / other
Never / Rarely / Sometimes / Often / Always</td></tr>
<tr><td>Original</td><td>F. STORE ACCESS
<table>
<tr><th>Please indicate whether the following statements are true of the store <u>where you usually buy groceries</u>.</th><th>Yes</th><th>No</th><th>Not applicable</th></tr>
<tr><td>1. Close to location of my employment</td><td>○</td><td>○</td><td>○</td></tr>
<tr><td>2. Close to my child's school</td><td>○</td><td>○</td><td>○</td></tr>
<tr><td>3. Close to my home</td><td>○</td><td>○</td><td>○</td></tr>
</table></td></tr>
<tr><td>Adapted</td><td>(removed)</td></tr>
<tr><td>Original</td><td>G. FOOD SHOPPING
<table>
<tr><th>The following questions apply to the store <u>where you usually buy groceries</u>.</th><th>Strongly disagree</th><th>Somewhat disagree</th><th>Neutral</th><th>Somewhat agree</th><th>Strongly agree</th></tr>
<tr><td>1. Low-fat foods cost too much.</td><td>○</td><td>○</td><td>○</td><td>○</td><td>○</td></tr>
<tr><td>2. There is a large selection of fresh fruits and vegetables.</td><td>○</td><td>○</td><td>○</td><td>○</td><td>○</td></tr>
<tr><td>3. There is a large selection of low-fat products available.</td><td>○</td><td>○</td><td>○</td><td>○</td><td>○</td></tr>
<tr><td>4. The condition of fresh fruits and vegetables is poor.</td><td>○</td><td>○</td><td>○</td><td>○</td><td>○</td></tr>
<tr><td>5. Fruits and vegetables cost too much.</td><td>○</td><td>○</td><td>○</td><td>○</td><td>○</td></tr>
</table></td></tr>
<tr><td>Adapted</td><td>În locul din care cumpărați cel mai frecvent alimente:
Mâncărurile sănătoase sunt prea costisitoare / Există o varietate bogată de fructe și legume proaspete / Există o varietate bogată de mâncăruri sănătoase / Fructele și legumele proaspete sunt într-o condiție precară / Fructele și legumele proaspete sunt prea costisitoare
Total Dezacord / Dezacord / Indiferent / Acord / Acord total</td></tr>
<tr><td>Backward</td><td>In the store where you most frequently buy food:
Healthy food is too expensive / there is a rich variety of fresh fruits and vegetables / there is a rich variety of healthy food / fruits and vegetables/fresh vegetables are in poor condition / fresh fruits and vegetables are too expensive
Total disagreement / disagreement / indifferent / Agreement / total agreement</td></tr>
</table>

<table>
<tr><td>Original</td><td>
H. YOUR CHILD'S ELECTRONICS
<table>
<tr><th>Please indicate whether the following are <u>in your child's bedroom</u>.</th><th>Yes</th><th>No</th></tr>
<tr><td>1. TV</td><td>○</td><td>○</td></tr>
<tr><td>2. Computer</td><td>○</td><td>○</td></tr>
<tr><td>3. Video game system (non-hand held; Playstation, Xbox, etc.)</td><td>○</td><td>○</td></tr>
<tr><th>Does your child have the following items for his/her own use?</th><th></th><th></th></tr>
<tr><td>4. Cell phone or 2-way radio</td><td>○</td><td>○</td></tr>
<tr><td>5. Hand-held videogame players (Game Boy, Sony PSP, etc.)</td><td>○</td><td>○</td></tr>
<tr><td>6. Music systems (Ipod, stereo, radio, etc.)</td><td>○</td><td>○</td></tr>
</table>
</td></tr>
<tr><td>Forward</td><td>Vă rugăm marcați care dintre următoarele electronice se găsesc în dormitorul copilului
Televizor / Calculator/laptop / Sistem de jocuri video (Playstation, Xbox, Nintendo, etc.)
Da/Nu
Vă rugăm marcați care dintre următoarele electronice se află la dispoziția sau în posesia copilului:
Telefon mobil / Sistem de jocuri portabil / (Game-Boy, Playstation portabil, etc.) / Sistem de redare muzică (mp3-player, ipod, etc.) / Tabletă electronica
Da/Nu</td></tr>
<tr><td>Backward</td><td>Please mark which of the following electronics are in your child's bedroom TV / computer / laptop / video game system (Playstation, Xbox, Nintendo, etc.)
Yes / No
Please mark which of the following electronics is available to or in the child's possession:
Mobile phone / portable gaming system /(Game-Boy, PlayStation Portable, etc.) / music playback system (mp3-player, ipod, etc.) / electronic tablet
Yes / No</td></tr>
<tr><td>Original</td><td>
For the next two questions, please think about your child's activities over the past year.
I. PLAY EQUIPMENT
<table>
<tr><th>How often during the past year has your child used these items at or around home (or in a common apartment area)?</th><th>Not available (Don't have)</th><th>Available but never use</th><th>Once a month or less</th><th>Once every other week</th><th>Once a week or more</th></tr>
<tr><td>1. Bike</td><td>○</td><td>○</td><td>○</td><td>○</td><td>○</td></tr>
<tr><td>2. Basketball hoop</td><td>○</td><td>○</td><td>○</td><td>○</td><td>○</td></tr>
<tr><td>3. Jump rope</td><td>○</td><td>○</td><td>○</td><td>○</td><td>○</td></tr>
<tr><td>4. Active video games (e.g., with dance pad, Wii, etc)</td><td>○</td><td>○</td><td>○</td><td>○</td><td>○</td></tr>
<tr><td>5. Sports equipment (like ball, racquets, bats, sticks)</td><td>○</td><td>○</td><td>○</td><td>○</td><td>○</td></tr>
<tr><td>6. Swimming pool</td><td>○</td><td>○</td><td>○</td><td>○</td><td>○</td></tr>
<tr><td>7. Roller skates, skateboard, scooter</td><td>○</td><td>○</td><td>○</td><td>○</td><td>○</td></tr>
<tr><td>8. Fixed play equipment (e.g., swing set, playhouse, jungle gym)</td><td>○</td><td>○</td><td>○</td><td>○</td><td>○</td></tr>
</table>
</td></tr>
<tr><td>Adapted</td><td>Vă rugăm marcați cât de frecvent a utilizat copilul aflat în grija dumneavoastră pe parcursul ultimului an următoarele obiecte/facilități
Bicicletă / Coș de baschet / Coardă de sărituri / Jocuri video active (de ex. care implică dans, mișcare) / Echipamente sportive (minge de fotbal sau baschet, rachete de tenis, etc.) / Role, skateboard, trotinetă (nu electrică) / Piscină / Echipamente de joacă fixe (tobogane, leagăne, masă de ping-pong, etc.)
Nu se aplică/nu are la dispoziție / Are la dispoziție, dar nu a folosit deloc / O dată pe lună sau mai puțin / O dată la două săptămâni / Săptămânal sau mai des</td></tr>
<tr><td>Backward</td><td>Please mark how frequently the child in your care has used the following objects/facilities during the last year
Bicycle / basketball hoop / jumping rope / active video games (eg. dance, movement) / sports equipment (football or basketball, tennis rackets, etc.) / Rollers, skateboard, scooter (not electric) / swimming pool / fixed play equipment (slides, swings, ping-pong table, etc.)
Not applicable/not available / has available but has not used at all / once a month or less / once every two weeks / Weekly or more often</td></tr>
</table>

<table>
<tr><td>Original</td><td>
J. PLACES FOR YOUR CHILD'S PHYSICAL ACTIVITY
<table>
<tr><th>How often during the past year has your child been <u>physically active</u> (including active play) in the following places?</th><th>Never</th><th>Once a month or less</th><th>Once every other week</th><th>Once a week</th><th>2 or 3 times/ week</th><th>4 times/ week or more</th></tr>
<tr><td>1. Inside your home</td><td>○</td><td>○</td><td>○</td><td>○</td><td>○</td><td>○</td></tr>
<tr><td>2. In your yard or common area or in your driveway</td><td>○</td><td>○</td><td>○</td><td>○</td><td>○</td><td>○</td></tr>
<tr><td>3. At a neighbor's house, yard, or driveway</td><td>○</td><td>○</td><td>○</td><td>○</td><td>○</td><td>○</td></tr>
<tr><td>4. In a local street, sidewalk, or vacant lot/field</td><td>○</td><td>○</td><td>○</td><td>○</td><td>○</td><td>○</td></tr>
<tr><td>5. Indoor recreation or exercise facility (public or private; e.g., YMCA/Boys & Girls Club)</td><td>○</td><td>○</td><td>○</td><td>○</td><td>○</td><td>○</td></tr>
<tr><td>6. Beach, lake, river, or creek</td><td>○</td><td>○</td><td>○</td><td>○</td><td>○</td><td>○</td></tr>
<tr><td>7. Bike/hiking/walking trails, paths</td><td>○</td><td>○</td><td>○</td><td>○</td><td>○</td><td>○</td></tr>
<tr><td>8. Basketball court</td><td>○</td><td>○</td><td>○</td><td>○</td><td>○</td><td>○</td></tr>
<tr><td>9. Other playing fields/courts (like football, softball, tennis)</td><td>○</td><td>○</td><td>○</td><td>○</td><td>○</td><td>○</td></tr>
<tr><td>10. Small public park or playground</td><td>○</td><td>○</td><td>○</td><td>○</td><td>○</td><td>○</td></tr>
<tr><td>11. Large public park</td><td>○</td><td>○</td><td>○</td><td>○</td><td>○</td><td>○</td></tr>
<tr><td>12. Public open space that is not a park</td><td>○</td><td>○</td><td>○</td><td>○</td><td>○</td><td>○</td></tr>
<tr><td>13. School grounds (during non-school hours)</td><td>○</td><td>○</td><td>○</td><td>○</td><td>○</td><td>○</td></tr>
</table>
</td></tr>
<tr><td>Forward</td><td>Vă rugăm marcați cât de frecvent a avut copilul aflat în grija dumneavoastră activitate fizică în următoarele locații pe parcursul ultimului an
Acasă / În curte / Acasă la un vecin sau prieten / Pe o stradă din apropierea domiciliului / Sală de sport sau complexe de recreere în interior / La ștrand / Trasee de drumeție / Terenuri de joacă (baschet, fotbal, tenis etc.) / Parcuri mici de cartier / Parcuri publice mari / Școală (extracurricular) / Alte spații publice
Niciodată / O dată pe lună sau mai puțin / O dată la două săptămâni / O dată pe săptămână / De 2-3 ori pe săptămână / De 4 ori pe săptămână sau mai des</td></tr>
<tr><td>Backward</td><td>Please mark how frequently your child has had physical activity at the following locations during the past year
Home / in the yard / home of a neighbor or friend / on a street near the home / gym or indoor recreation complexes / at the swimming pool / hiking trails / playgrounds (basketball, football, tennis, etc.) / small neighborhood parks / large public parks / School (extracurricular) / other public spaces
Never / once a month or less / once every two weeks / once a week / 2-3 times a Week / 4 times a week or more often</td></tr>
</table>

Original

K. GETTING AROUND IN YOUR NEIGHBORHOOD

Please select the answer that best applies to you and your neighborhood. Within walking distance means within a 10-15 minute walk from your home.

	Strongly disagree	Somewhat disagree	Somewhat agree	Strongly agree
1. There are shops, stores, markets, and places to buy things I need within easy walking distance of my home/house.	○	○	○	○
2. There is a bus, subway, or train stop within walking distance from my home.	○	○	○	○
3. There are sidewalks on most streets.	○	○	○	○
4. There are NOT many dead end streets.	○	○	○	○
5. There are many different routes for getting from place to place.	○	○	○	○
6. There is a high crime rate.	○	○	○	○
7. The speed of traffic on most streets is usually slow (30 mph or less).	○	○	○	○
8. Most drivers go faster than the posted speed limits.	○	○	○	○
9. There are many interesting things to look at while walking in my neighborhood.	○	○	○	○
10. The traffic makes it difficult or unpleasant for my child to walk.	○	○	○	○
11. Streets have good lighting at night.	○	○	○	○
12. There are crosswalks and signals on busy streets.	○	○	○	○
13. There are many places to go within easy walking distance of my home.	○	○	○	○
14. I'm afraid of my child being taken or hurt by a stranger on local streets.	○	○	○	○
15. I'm afraid of my child being taken or hurt by a stranger in my yard, driveway, or common area.	○	○	○	○
16. I'm afraid of my child being taken or hurt by a stranger in a local park.	○	○	○	○
17. I'm afraid of my child being taken or hurt by a known "bad" person (adult or child) in my neighborhood.	○	○	○	○

Adapted

(removed)

Original

L. DISTANCE TO LOCATIONS

About how long would it take <u>you</u> to walk from your home to the nearest places listed below? Please select the time it would take you to walk to each place, regardless of whether you/your child go there.

	1-5 min	6-10 min	11-20 min	21-30 min	31+ min	Don't know
1. Convenience/corner store/small grocery store/bodega	○	○	○	○	○	○
2. Supermarket	○	○	○	○	○	○
3. Fast food restaurant	○	○	○	○	○	○
4. Non-fast food restaurant	○	○	○	○	○	○
5. Indoor recreation or exercise facility (public or private; e.g., YMCA/Boys & Girls Club)	○	○	○	○	○	○
6. Beach, lake, river, or creek	○	○	○	○	○	○
7. Bike/hiking/walking trails, paths	○	○	○	○	○	○
8. Basketball court (including half-court)	○	○	○	○	○	○
9. Other playing fields/courts (like soccer, football, softball, tennis, skate park, etc.)	○	○	○	○	○	○
10. Small public park	○	○	○	○	○	○
11. Large public park	○	○	○	○	○	○
12. Public playground with equipment	○	○	○	○	○	○
13. School with recreation facilities open to the public	○	○	○	○	○	○

Forward

Vă rugăm marcați cât ar dura aproximativ pentru a ajunge în mers pe jos până la urmatoarele facilități de la domiciliul copilului aflat în grija dumneavoastră:

Magazin mic de cartier / Supermarket / Restaurant fast-food / Sală de sport / Ștrand / Terenuri de joacă (baschet, tenis, fotbal, etc.) / Parcuri mici de cartier / Parcuri publice mari / Spații publice cu echipamente de sport

1-5 minute / 6-10 minute / 11-20 minute / 21-30 minute / Peste 30 de minute / Nu știu

Backward

Please mark how long it would take approximately to walk to the following facilities at the home of the child in your care:

Small neighborhood store / Supermarket / fast food restaurant / gym / swimming pool / playgrounds (basketball, tennis, football, etc.) / small neighborhood parks / large public parks / public spaces with sports equipment

1-5 minutes / 6-10 minutes / 11-20 minutes / 21-30 minutes / over 30 minutes / I don't know

<table>
<tr><td>Original</td><td>M. FAMILY
During a typical week, how often do you or another adult in the household:
<table>
<tr><th></th><th>Never</th><th>1-2 days</th><th>3-4 days</th><th>5-6 days</th><th>Every day</th></tr>
<tr><td>1. Watch your child participate in physical activity or sports</td><td>○</td><td>○</td><td>○</td><td>○</td><td>○</td></tr>
<tr><td>2. Encourage your child to do sports or physical activity</td><td>○</td><td>○</td><td>○</td><td>○</td><td>○</td></tr>
<tr><td>3. Provide transport to a place where your child can do physical activity or play sports</td><td>○</td><td>○</td><td>○</td><td>○</td><td>○</td></tr>
<tr><td>4. Do a physical activity or play sports with your child</td><td>○</td><td>○</td><td>○</td><td>○</td><td>○</td></tr>
</table></td></tr>
<tr><td>Adapted</td><td>În timpul unei săptămâni uzuale, cât de frecvent dumneavoastră sau un alt adult din familia copilului
Urmăriți copilul în timp ce practică activități fizice / Încurajați copilul practice activități fizice / Transportați copilul către un loc în care desfășoară activități fizice / Practicați o activitate fizică împreună cu copilul
Niciodată / 1-2 zile pe săptămână / 3-4 zile pe săptămână / 5-6 zile pe săptămână / Zilnic</td></tr>
<tr><td>Backward</td><td>During a typical week, how frequently do you or another adult in the child's family
Watch the child while practicing physical activities / encourage the child to practice physical activities / Transport the child to a place where they are doing physical activity / practice a physical activity with the child
Never / 1-2 days a week / 3-4 days a week / 5-6 days a week / Daily</td></tr>
<tr><td>Adapted question (WHO-COSI)</td><td>Vă rugăm marcați dacă mama copilului suferă sau a suferit de vreuna dintre aceste afecțiuni sau dacă a fost vreodată tratată pentru vreuna din ele:
Hipertensiune arterială (valori crescute ale tensiunii arteriale) / Diabet Zaharat / Obezitate / Cardiopatie ischemică / Infarct miocardic / Accident vascular cerebral / Arteriopatie cronică obliterantă a membrelor inferioare (îngustarea arterelor de la picioare) / Insuficiență cardiacă / Hipercolesterolemie (valori crescute ale colesterolului în sânge) / Hipertrigliceridemie (valori crescute ale trigliceridelor în sânge)</td></tr>
<tr><td>Translation</td><td>Please mark if the child's mother suffers or has suffered from any of these conditions, or if she has ever been treated for any of them:
Hypertension (high blood pressure) / Diabetes mellitus / Obesity / Ischaemic heart disease / Myocardial infarction / Stroke / Chronic obliterating arteriopathy of the lower limbs (narrowing of the arteries in the legs) / Heart failure / Hypercholesterolemia (high blood cholesterol) / Hypertriglyceridemia (high triglycerides in the blood)</td></tr>
<tr><td>Adapted question (WHO-COSI)</td><td>Vă rugăm marcați dacă tatăl copilului suferă sau a suferit de vreuna dintre aceste afecțiuni sau dacă a fost vreodată tratată pentru vreuna din ele:
Hipertensiune arterială (valori crescute ale tensiunii arteriale) / Diabet Zaharat / Obezitate / Cardiopatie ischemică / Infarct miocardic / Accident vascular cerebral / Arteriopatie cronică obliterantă a membrelor inferioare (îngustarea arterelor de la picioare) / Insuficiență cardiacă / Hipercolesterolemie (valori crescute ale colesterolului în sânge) / Hipertrigliceridemie (valori crescute ale trigliceridelor în sânge)</td></tr>
<tr><td>Translation</td><td>Please mark if the child's father suffers or has suffered from any of these conditions, or if he has ever been treated for any of them:
Hypertension (high blood pressure) / Diabetes mellitus / Obesity / Ischaemic heart disease / Myocardial infarction / Stroke / Chronic obliterating arteriopathy of the lower limbs (narrowing of the arteries in the legs) / Heart failure / Hypercholesterolemia (high blood cholesterol) / Hypertriglyceridemia (high triglycerides in the blood)</td></tr>
</table>

Table S2 – *Changelogs for the modifications made to the questionnaires utilized*

Questionnaire	Changelog
ISCOLE Diet and Lifestyle Questionnaire	Removed ″For the questions on this page, please tell about what you did last week.″ and instead mentioned the timeframe for the required answer within each question.<br>Added questions for the participant's name and age for easier record keeping<br>Made a clear distinction between electric and non-propelled ones<br>Added a follow-up question requesting further details in case the respondent an-swered ″other″ regarding how they travelled the main part of their journey to school<br>For the agreement scale used in the student questionnaire, we have decided to ex-plicitly name each of the response categories from 1 to 5 (1 = if you completely disagree with the statement, 2 = if you disagree with the statement, 3 = if you are undecided about the statement, 4 = if you agree with the statement, 5 = if you fully agree with the statement).<br>For questions 13–20 we replaced ″ I can″ with ″ I choose to″ or ″ I prefer to″ where appropriate in order to better reflect the nuances of these questions.<br>We added ″and motivation″ next to ″coordination″ for question 19 to expand its meaning.<br>Changed the answer options for questions regarding length of sleep to better re-flect the amount of time<br>Changed the hour format to 24h instead of 12h AM/PM<br>Added a question inquiring whether or not the participant had a computer in their bedroom, in addition to the one related to a television set<br>Added some options about food choices that are frequently consumed in Romania (i.e., sunflower seeds) and created a distinction between roasted and salted nuts and unroasted and unsalted nuts due to the different dietary profiles of these food types.<br>Removed the option ″Never″ in the final version of the questionnaire for questions re-garding dietary habits due to perceived overlap with the option ″less than once a week″ upon translation.<br>Created separate questions for breakfast consumption (school days vs. weekends)<br>Added an extra option for the final question of the diet and lifestyle questionnaire regarding the perception of health to provide more balanced options.
ISCOLE Demographic and Family Health Questionnaire	Merged the ISCOLE Demographic and Family Health Questionnaire, the Child Feeding Questionnaire and the elements included from the ISCOLE Neighbourhood and Family Environment Questionnaire into one single Questionnaire entitled "Ques-tionnaire for parents/guardians—Obesity study".<br>Added a short introduction describing the tasks to be completed within the ques-tionnaire and data management<br>Removed section titles<br>Added a description for the course of action for parents with multiple children enrolled in the study (i.e., filling out a questionnaire for each child)<br>Removed questions regarding address and instead added a question regarding the general environment in which the child's home is situated (urban vs. rural) in order to simplify data interpretation<br>Added a question regarding the nature of the relationship between the responder and the child included in the study—similar to the COSI questionnaire<br>Removed question regarding the age in years of the participating child, keeping only date of birth which was verified according to personal information contained in the school registry<br>Removed question regarding the ethnicity of the child—this data was retrieved from school records<br>Transformed response options for question regarding siblings into a tick-mark-based response for more explicit data regarding sibling gender and easier future implementation in web-based response settings<br>Added queries regarding sibling age, weight and height

	Added a question regarding the child's previous school<br>Added a question regarding the child's grade for easier book-keeping<br>Removed question regarding child length at birth due to previously described poor recall of this parameter in previous studies<br>Transformed the question regarding length of pregnancy into a series of questions in order to streamline responses whereby participants first selected whether or not the child was born preterm, at term or post-term and followed up with the precise length of pregnancy only for children not born at term.<br>Transformed the question regarding formula feeding into a series of questions in order to streamline responses whereby participants first selected whether or not the child was formula fed and followed up with questions regarding the initiation and stopping of formula feeding where applicable<br>Simplified the question regarding number of cohabitants of the child, in a similar manner to the COSI questionnaire<br>Question regarding family income was simplified and treated as the perception of the respondent—in a similar manner to the COSI questionnaire<br>Removed questions regarding television service and internet type due to particu-lar characteristics of the market regarding these services (i.e., ubiquity of optical fi-ber-based internet and television service providers)<br>Adapted response options of maternal and paternal education to correspond to ISCED levels, similar to the COSI questionnaire<br>Adapted response options of working hours to local working environment and legislation<br>Removed question regarding adoption—adopted children were excluded from the study<br>Created separate queries for the age, weight, and height of the parents
Child Feeding Questionnaire	Questions regarding monitoring were placed immediately after those concerning perceived responsibility and the same scale was used for these questions due to providing a more streamlined survey and a more balanced translation.<br>Answers for perceived weight questions were shifted towards the spectrum of ex-cess weight, due to several factors. One would be the relative rarity of what would be defined as "markedly underweight" as opposed to "extreme obesity". Second is due to the nature of the translation whereby "overweight" translates to "supraponderе". Although this term is defined by specific BMI intervals, this option may have not been suitable for less knowledgeable participants who may have been unaware of the pre-cise definitions involved, while others may have answered with higher precision. By extension, "markedly overweight" would have translated to "supraponderе marcată", a term which may have created confusion in a similar manner as described previously. In light of these nuances the decision was made to provide the options "supraponderе" (overweight), "obezitate" (obesity) and "obezitate extrema" (extreme obesity) to offer an easier qualitative interpretation of weight status for the participants.<br>Periods for perceived weight question were simplified for the adults to include only "childhood", "adolescence", and "present" while the periods for children were adapted to the timeframe corresponding to local education structures (primary clas-ses/Middle School/High School—whereby the last category coincided with present status).<br>Questions 14 and 16 regarding concern were merged into one question and adapted due to several reasons. Firstly, there is considerable overlap between question 14 and questions 17–19, 23, 29–31 as well as a newly added question that was placed after question 28 of the original questionnaire (see below). In addition, the difference in nuance between "concern" and the need to keep track, monitor or regulate food intake would have been lost in translation. Finally, while question 16 made reference only to future weight status, a more general approach regarding this aspect (referring to the child's weight in general, both currently and in the future) was deemed more appro-priate, particularly in the scenario where question 14 was omitted.

	The answers to questions regarding concern were modified into a 5-point Likert scale ranging from "not at all worried" (or concerned) to "very worried" (or con-cerned) without explicit mention of each point on the scale due to the difficulty of ob-taining a clear grading for levels of concern upon translation.<br>Added "sugary juices" along with sweets as well as "chocolate" within this de-scription as a definitory for hypercaloric carbohydrate-laden food items<br>A different scale was used for questions 21 and 22 due to these inquiries implying frequency of a behaviour rather than agreement to them (as opposed to questions 17, 18, 23, 24).<br>For question 23 the term "junk food" was replaced with "unhealthy food" during translation<br>Question 24 was removed due to significant overlap with question 19 upon trans-lation.<br>A question was added after question 28 of the initial questionnaire with a similar construction to question 28, but enquiring about the situation of overeating rather than insufficient food consumption.<br>Consequently, questions 21 and 22 were moved after the newly added question in the final questionnaire to streamline the survey, particularly due to the fact that ques-tions 17, 18, and 23 implemented the same response scale as questions 25–28.
ISCOLE Neighborhood and Home Environment Questionnaire	Sections A (neighborhood cohesion), B (neighbors and friends), C (neighborhood response), and K (getting around in your neighborhood) were omitted due to exceeding the scope of our study.<br>Created a distinction between roasted and salted nuts and unroasted and unsalted nuts due to different dietary profiles of these food types<br>Provided some examples of hypermarket chains and added "local producers" as an option<br>Removed section F due to significant overlap between questions in this section and section L and limited significance for our study<br>Replaced "low-fat foods" with the more general term "healthy foods" in section G<br>Added "electronic tablet" as option in section H<br>Added "pool" as option in section J and created a single option for both basketball courts and other courts and added the option for "other public spaces"<br>In section L: removed "non-fast food restaurant" to avoid confusion with fast-food restaurants, removed "school with recreation facilities open to the public" due to variable school policies and lack of a legal context for access of non-students on school grounds, replaced "beach, lake, river or creek" with "swimming pool", due to geographic specifics pertaining to our study, removed "bike/hiking/walking trails, paths" due to their general ubiquity in our geographic area, created a single option for both basketball courts and other courts, removed "school or recreation facilities open to the public" due to perceived redundancy.

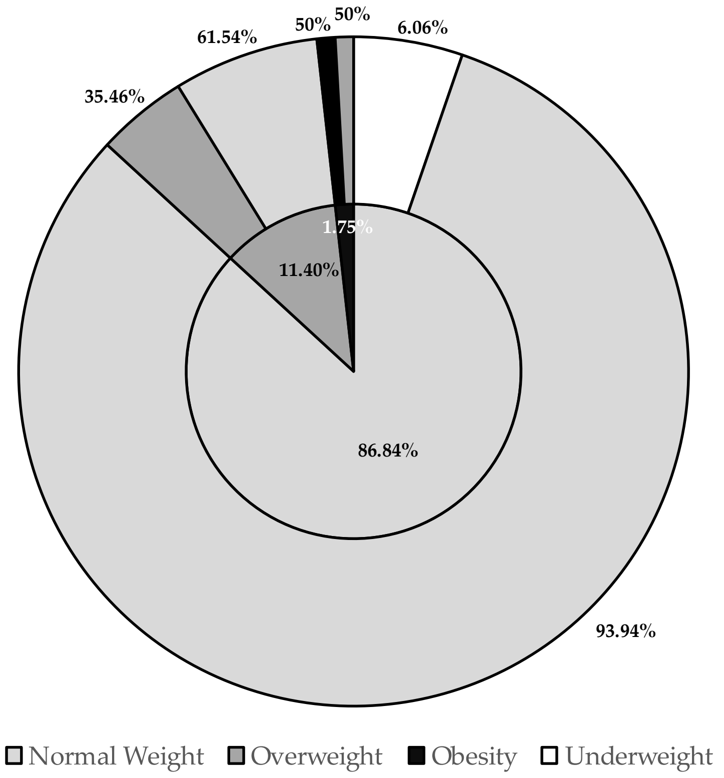

Figure S1. Parental perception of child weight category. Calculated student weight category based on BMI at the time of data collection is shown in the inner circle (as a percentage of the 114 enrolled students) and parent-perceived student weight category is shown in the outer circle (as a percentage of calculated weight category).

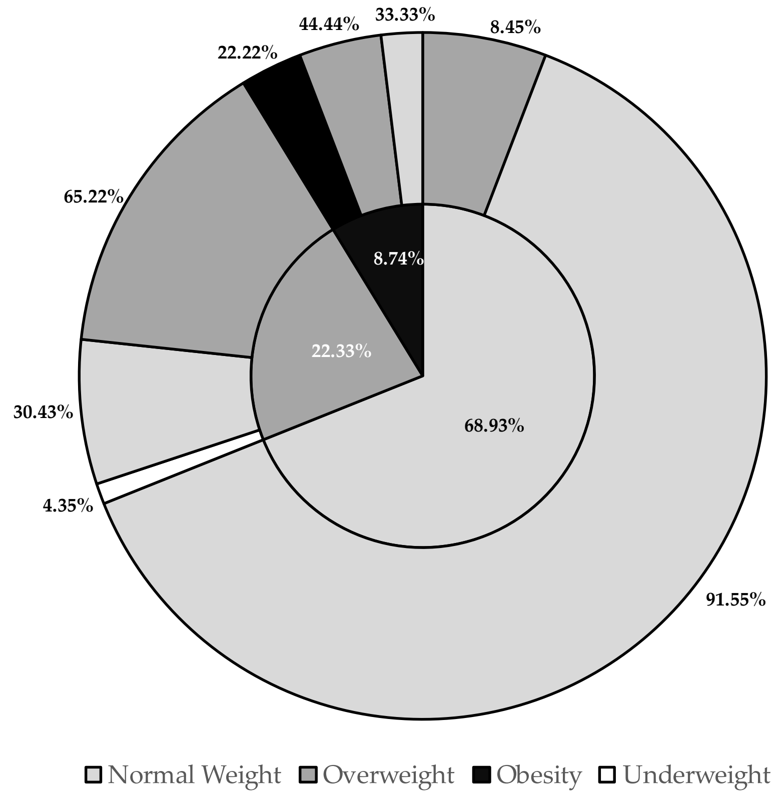

Figure S2. Parental self-perception of weight category. Calculated parent weight category based on BMI is shown in the inner circle (as a percentage of the 103 respondent mothers) and self-perceived weight category is shown in the outer circle (as a percentage of calculated weight category).

Table S3. Student average marks at the end of the year across various categories

Tested Variable	Categories	Number of Students	Average of Grades for 9th Grade Students			*p*-Value
			Mean (95% CI)	SD/IQR	MIN–MAX	
Weekday sleep duration	< 8 h	39	9.37 (9.25–9.48)	0.35/0.47	8.33–10	0.038
	≥ 8 h	20	9.56 (9.42–9.69)	0.3/0.45	8.82–9.95	
Estimated family income (missing = 1)	High	13	9.39 (9.28–9.49)	0.35/0.44	8.33–9.95	0.105
	Low or moderate	45	9.56 (9.41–9.71)	0.25/0.57	9.26–10	
Maternal higher education	No (ISCED ≤ 4)	23	9.51 (9.38–9.63)	0.3/0.54	9.11–10	0.171
	Yes (ISCED > 4)	36	9.38 (9.26–9.5)	0.36/0.4	8.33–9.95	
Paternal higher education	No (ISCED ≤ 4)	24	9.52 (9.4–9.63)	0.27/0.47	9.11–10	0.105
	Yes (ISCED > 4)	35	9.37 (9.24–9.5)	0.37/0.53	8.33–9.95	
**Tested Variable**	**Categories**	**Number of Students**	**Average of Grades for 10th Grade Students**			***p*-Value**
			**Mean (95% CI)**	**SD/IQR**	**MIN–MAX**	
Weekday sleep duration	<8 h	43	9.35 (9.22–9.47)	0.4/0.71	8.5–10	0.379
	≥8 h	12	9.46 (9.25–9.67)	0.33/0.38	8.85–10	
Estimated family income	High	19	9.45 (9.22–9.69)	0.33/0.93	8.5–10	0.343
	Low or moderate	35	9.33 (9.22–9.45)	0.33/0.39	8.68–10	
Maternal higher education	No (ISCED ≤ 4)	18	9.39 (9.21–9.57)	0.36/0.54	8.84–10	0.812
	Yes (ISCED > 4)	37	9.36 (9.23–9.5)	0.4/0.66	8.5–10	
Paternal higher education	No (ISCED ≤ 4)	23	9.38 (9.24–9.53)	0.33/0.43	8.68–9.95	0.877
	Yes (ISCED > 4)	32	9.37 (9.21–9.52)	0.42/0.78	8.5–10	

ISCED—International Standard Classification of Education; 95%CI—95% confidence interval for the mean; StdDev—standard deviation; IQR—interquartile range; MIN—minimum observed value; MAX—maximum observed value.
